# Prediction of Energy Storage Performance in Polymer Composites Using High‐Throughput Stochastic Breakdown Simulation and Machine Learning

**DOI:** 10.1002/advs.202105773

**Published:** 2022-04-10

**Authors:** Dong Yue, Yu Feng, Xiao‐Xu Liu, Jing‐Hua Yin, Wen‐Chao Zhang, Hai Guo, Bo Su, Qing‐Quan Lei

**Affiliations:** ^1^ School of Materials Science and Chemical Engineering Harbin University of Science and Technology Harbin 150080 China; ^2^ Key Laboratory of Engineering Dielectrics and Its Application Ministry of Education Harbin University of Science and Technology Harbin 150080 China; ^3^ School of Materials Science and Engineering Shaanxi University of Science and Technology Xi'an 710021 China; ^4^ College of Computer Science and Technology Dalian Minzu University Dalian 116650 China

**Keywords:** breakdown strength, energy storage density, machine learning, polymer‐based composites, stochastic breakdown simulation

## Abstract

Polymer dielectric capacitors are widely utilized in pulse power devices owing to their high power density. Because of the low dielectric constants of pure polymers, inorganic fillers are needed to improve their properties. The size and dielectric properties of fillers will affect the dielectric breakdown of polymer‐based composites. However, the effect of fillers on breakdown strength cannot be completely obtained through experiments alone. In this paper, three of the most important variables affecting the breakdown strength of polymer‐based composites are considered: the filler dielectric constants, filler sizes, and filler contents. High‐throughput stochastic breakdown simulation is performed on 504 groups of data, and the simulation results are used as the machine learning database to obtain the breakdown strength prediction of polymer‐based composites. Combined with the classical dielectric prediction formula, the energy storage density prediction of polymer‐based composites is obtained. The accuracy of the prediction is verified by the directional experiments, including dielectric constant and breakdown strength. This work provides insight into the design and fabrication of polymer‐based composites with high energy density for capacitive energy storage applications.

## Graphical abstract

The effects of single variable and multivariable coupling on the breakdown strength of polymer‐based composites are studied by high‐throughput stochastic breakdown simulation, and a machine learning database is established to obtain breakdown prediction. A universal energy storage density prediction is obtained by combining the breakdown prediction with the classical dielectric formula, and the predictions' accuracy is verified.

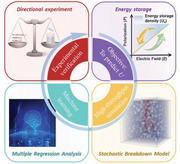

## 1 Introduction

Currently, energy storage systems have three main categories: fuel cells, electrochemical capacitors, and dielectric capacitors.^[^ ^1^, ^2^, ^3^, ^4^ ^]^ Compared with the fuel cells and electrochemical capacitors, the dielectric capacitors possess the significant advantage of high power energy density (>10^8^ W kg^−1^) and fast charge/discharge speed (<0.01 s), which has been widely applied in high power/pulse power technologies, such as motor drive, mobile power system, and space vehicle power supply.^[^ ^5^, ^6^, ^7^, ^8^ ^]^ Energy storage density is an important factor in the polymer dielectric capacitors.^[^ ^9^, ^10^, ^11^ ^]^ Generally, the energy storage density (*U*) of dielectrics can be approximately predicted via following expression as *U* = 1/2*ε*_r_ *ε*_0_ *E* ^2^, where *ε*_r_ is the relative dielectric constant, *ε*_0_ is the vacuum dielectric constant (8.85 × 10^−12^ F m^−1^) and *E* is the applied electric field. Figure S1 (Supporting Information) can be seen more intuitively that *U* is governed by two factors of polarization (*P*) and *E* in the Supporting Information, the *P*’s strength is closely related to the *ε*_r_. Hence, both high *P* and *E* are desirable for high *U*.

There have been intensive experimental activities to synthesize polymer‐based composites with enhanced *U*, most of which largely employed trial‐and‐error and intuition‐driven methods, there are few reports on the prediction of *U*. There are a lot of reports for *ε*_r_ prediction,^[^ ^12^, ^13^, ^14^ ^]^ but few reports predict the dielectric breakdown strength (*E*_b_), even if *E* is the applied electrical field and a known quantity. When an electric field is applied, the field distribution inside a polymer‐based composite is complex. The electric breakdown represents the irreversible damage caused by avalanche multiplications of free charges due to high locally induced electric field. Therefore, when carriers acquire sufficient kinetic energy in the collision with the polymer matrix to give a high probability of ionization, more carriers are generated.^[^ ^15^, ^16^, ^17^, ^18^ ^]^ A number of possible dielectric breakdown mechanisms and factors of polymer‐based composites have been proposed, including filler *ε*_r_, filler size (*d*), and filler content (*v*), but these proposals tend to be limited to single variable, while few of them take into account multiple variables.^[^ ^19^, ^20^, ^21^, ^22^ ^]^ Hence, building sensible multiple variables links to gain fundamental insights on dielectric breakdown behaviors is instructive to innovative design of high *U* polymer‐based composites.

In recent years, the breakdown simulation (including phase‐field and stochastic breakdown simulation) and machine learning have been widely applied in energy storage dielectrics.^[^ ^23^, ^24^, ^25^, ^26^ ^]^ The phase‐field method is based on the kinetics of mesoscale inhomogeneous systems and can fundamentally understand and predict the microstructure evolution of composites under different electric field strengths and their relationship with the polymer‐based composites' behavior.^[^ ^27^, ^28^, ^29^ ^]^ The stochastic breakdown model is adopted to simulate the breakdown path evolution in a way and with a purpose similar to the phase‐field model, which can solve the electric field distribution of composites.^[^ ^30^, ^31^ ^]^ The difference is that the stochastic breakdown model considers the stochasticity of breakdown by calculating the electric field distribution, which is more consistent with the actual physical breakdown process in the composite.^[^ ^32^ ^]^ The machine learning can predict the performance parameters of composites by establishing linear or nonlinear mathematical model relationships among multiple variables.^[^ ^33^, ^34^ ^]^ In this work, we use a comprehensive stochastic breakdown model with three variables to investigate the filler effects on the breakdown process of polymer‐based composites, and the three most important factors, include filler *ε*_r_, *d*, and *v*. Using two representative polymers, polyimide (PI) and poly(vinylidene fluoride) (PVDF), the underlying physical mechanisms are analyzed by simulating the evolution of breakdown paths in polymer‐based composites with different *ε*_r_, *d*, and *v* of fillers. Then, by performing 504 groups of high‐throughput stochastic breakdown simulation results, used *ε*_r_, *d*, and *v* as variables, a database of bridging microstructures and *E*_b_ of polymer‐based composites is constructed as the database for machine learning. By combining the classical formula of *ε*_r_ with *E*_b_ prediction expression, *U* prediction expression is obtained. Finally, polyetherimide (PEI)/aluminum oxide (Al_2_O_3_) composites were prepared to validate the predictions, the microstructure characterization and performance tests of PEI/Al_2_O_3_ composites were carried out systematically. The *U* experiment results are basically consistent with the calculated results of *U* predictions, which also confirms the universality and accuracy of *U* predictions.

## 2 Results and Discussion

### 2.1 High‐Throughput Stochastic Breakdown Simulation of Polymer‐Based Composites

Although the breakdown simulation has been used frequently to predict the breakdown path development of polymer‐based composites, due to the diversification of models and approaches, it is necessary to verify the accuracy of a model before using it.^[^ ^35^, ^36^, ^37^, ^38^ ^]^ The stochastic breakdown simulation of the PI/Al_2_O_3_ composites (1, 3, 5, 7, 9 vol%) were carried out, the size of Al_2_O_3_ was set at 50 nm. The simulation results are close to the results in the published literature, the *E*_b_ of the PI/Al_2_O_3_ composites increases first and then decreases in Figure S2 (Supporting Information).^[^ ^39^ ^]^ Here, PI/silicon dioxide (SiO_2_) and PI/barium titanate (BaTiO_3_) composites are examples to study how the fillers affect the *E*_b_. As can be seen in Figure S3 (Supporting Information), SiO_2_, due to its characteristics of low *ε*_r_ and high *E*_b_, hindered the development of the breakdown path to a certain extent, while BaTiO_3_ has a high *ε*_r_, and the breakdown path easily passes through the fillers. The purpose of this work is to improve the *E*_b_ of polymer‐based composites, fillers with low *ε*_r_ were chosen for high‐throughput stochastic breakdown simulations.

**Figure 1:**
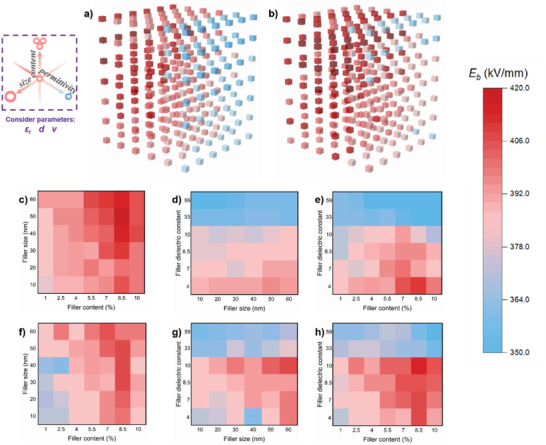
High‐throughput stochastic breakdown simulations. The breakdown strength (*E*_b_) of a) PI matrix b) PVDF matrix with three variables, i.e., the filler sizes (*d*) (from 10, 20, 30, 40, 50, 60 nm), the filler contents (*v*) (from 1, 2.5, 4, 5.5, 7, 8.5, 10 vol%), and the filler dielectric constants (*ε_r_*) (from 4, 7, 8.5, 10, 33, 59); 2D mapping of *E*_b_ of PI c–e) and PVDF f–h) as a function of two out of the three variable (*d*, *v*, *ε*_r_).

Here, two typical polymers, PI and PVDF, are selected as studying examples. It should be noted here that PVDF was selected for its high *ε*_r_ only, and its ferroelectric property was not considered, and *ε*_r_ of PI in polymer is low. The stochastic breakdown simulation results of the two polymers can be compared, the fillers are assumed to be randomly distributed and spherical. The effect of fillers on the breakdown process of polymer‐based composites was studied by means of stochastic breakdown simulation. Three parameters of the fillers, including the size *d*, content *v*, and dielectric constant *ε*_r_, are chosen as the parameters for the high‐throughput stochastic breakdown simulation. The ranges of *d*, *v*, and *ε*_r_ are chosen to be 10, 20, 30, 40, 50, 60 nm, 1, 2.5, 4, 5.5, 7, 8.5, 10 vol%, and 4 (SiO_2_), 7 (zinc oxide, ZnO), 8.5 (aluminum nitride (AlN)), 10 (Al_2_O_3_), 33 (zirconium dioxide (ZrO2)), 59 (titanium dioxide (TiO_2_)), respectively, which include fillers commonly used to enhance the *E*_b_ of polymer‐based composites, and note that the nonlinear dielectric properties of ZnO are not taken into consideration when ZnO is selected as a filler in this work. Most information on high‐throughput stochastic breakdown simulations can be found in Figures S4–S15 and Table S1 (Supporting Information). The results are shown in **Figure** **1**a,b, it is clearly seen that *d*, *v*, and *ε_r_* all have certain effects on the *E*_b_. It shows that the fillers with larger *d*, higher *v*, and lower *ε_r_* exhibit relatively higher *E*_b_ of polymer‐based composites, as indicated by the red region in Figure 1a,b. To clearly identify the trend from 3D database, the 2D mapping of *E*_b_ is shown as a function of two out of the three variables, Figure 1c–e corresponds to PI composites, and Figure 1f–h corresponds to PVDF composites. Figure 1c,f shows the dependence of *E*_b_ on *d* and *v*. Figure 1d,g shows the dependence of *E*_b_ on *d* and *ε*_r_. Figure 1e,h shows the dependence of *E*_b_ on *v* and *ε*_r_. These 2D mapping results indicate that larger *d* and higher *v* give rise to higher *E*_b_, but when the *v* is too high, the *E*_b_ decreases, especially for the fillers of large *d*. On the other hand, from the 2D mapping of PI composites, it shows a general trend that the fillers with larger *ε*_r_ exhibit relatively larger *E*_b_, while in PVDF composites, the *E*_b_ increases first and then decreases with the increase of *ε*_r_. All these trends show that each variable has its own way to exert its influence on the *E*_b_. Therefore, we studied the influence of each parameter on *E*_b_ by fixing other two parameters and only considering a single variable at a time.

**Figure 2:**
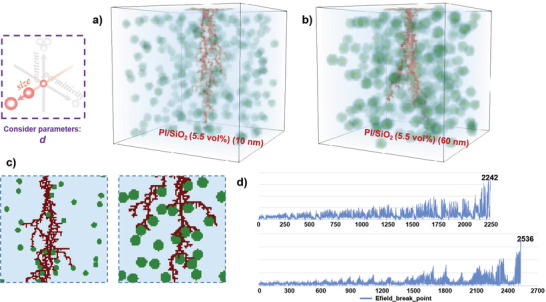
The 3D stochastic breakdown simulations of a) PI/SiO_2_ (5.5 vol%) (10 nm) and b) PI/SiO_2_ (5.5 vol%) (60 nm) composite. c) Local 2D cross‐sectional view of the breakdown paths of two polymer‐based composites. d) Iteration steps of the breakdown paths of two polymer‐based composites.

First, two 3D stochastic breakdown models of the polymer‐based composites with the *v* and *ε_r_* of the fixed fillers were established, only considering the *d* change, the PI/SiO_2_ (5.5 vol%) composites with 10 and 60 nm, as shown in **Figure** **2**a,b, respectively. It can be seen that at the same *v* and *ε*_r_, the breakdown paths of the polymer‐based composite with large filler *d* like a flourishing tree branch, which is more tortuous than that of the polymer‐based composite with small filler *d*. To display the local characteristics clearly, especially at the surface region of the fillers, the local 2D cross‐sectional view and the iteration steps of breakdown paths are plotted in Figure 2c,d, respectively. It can be clearly seen that due to the large *d* of the 60 nm fillers, the breakdown path required when the electric field is applied to the fillers is longer than that of the 10 nm fillers. Without considering the influence of *v* and *ε*_r_, a longer breakdown path will lead to a higher *E*_b_ of the polymer‐based composites. This phenomenon indicates the blocking effect to describe the role of fillers on delaying and hindering the propagation of the breakdown path.^[^ ^34^ ^]^ This also shows that in different shapes of the same fillers, nanosheets has a stronger blocking effect than nanowires and nanoparticles, and has a stronger ability to improve the *E*_b_ of polymer‐based composites.^[^ ^27^, ^29^, ^32^ ^]^

**Figure 3:**
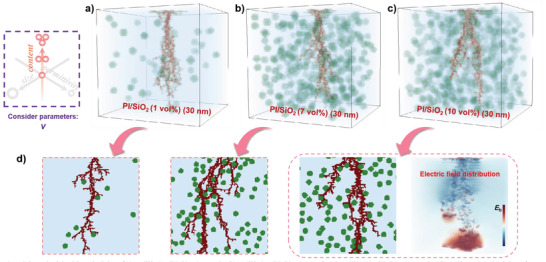
3D stochastic breakdown simulations of a) a PI/SiO_2_ (1 vol%) (30 nm) composite; b) a PI/SiO_2_ (7 vol%) (30 nm) composite; c) a PI/SiO_2_ (10 vol%) (30 nm) composite. d) Local 2D cross‐sectional views of breakdown paths of three different states and electric field distribution of the excessive filling state.

Then, fixed *d* and *ε*_r_, changing *v*, the impact of *v* on the breakdown path development processes is simulated. As illustrated in **Figure** **3**a–c, here we consider three kinds of *v* (1, 7, and 10 vol%) of the polymer‐based composites, which represent a small amount of filling, an appropriate amount of filling, and an excessive amount of filling, respectively. From the 3D stochastic breakdown simulations, with the increase of *v*, the breakdown path changes from a “single‐way” to a “branchy” shape, indicating that the breakdown path may be hindered. However, at higher *v*, it is impossible to distinguish breakdown path which is more complex. In order to better understand the effects of the *v* on the *E*_b_ of polymer‐based composites, the 2D cross‐sectional views at the front of the breakdown paths are drawn in Figure 3d. The results show that the behave of breakdown path like a “single‐way” in the small amount of filling, and the probability of obstruction is very low. At the appropriate amount of filling, the breakdown path obviously changes from a “single‐way” to a “branchy” shape, and in the situation of excessive filling level, the “branchy” breakdown path turns into a “single‐way,” which is due to the breakdown paths' recombined. In order to understand this phenomenon, we plotted the electric field distribution at the front of the breakdown path under the excessive filling state. Due to excessive filler loading, the filler agglomeration probability increase. The local electric field was inevitably distorted since filler agglomerated inside the composite. Fast‐forward slender breakdown channel along the agglomerations, and the *E*_b_ of the polymer‐based composites is reduced. Therefore, an appropriate *v* exists to optimize the *E*_b_ of the polymer‐based composites, which also depends on the *d* and *ε*_r_ of the fillers.

**Figure 4:**
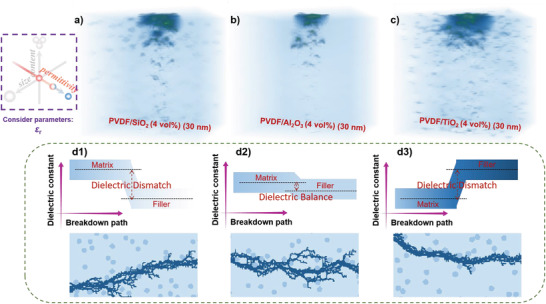
3D electric field distributions of a) a PVDF/SiO_2_ (4 vol%) (30 nm) composite; b) a PVDF/Al_2_O_3_ (4 vol%) (30 nm) composite; c) a PVDF/TiO_2_ (4 vol%) (30 nm) composite. d) A simple matrix‐filler dielectric matching diagram of polymer‐based composites with different fillers and corresponding 2D stochastic breakdown simulation breakdown path development.

Next, with fixed *d* and *v*, using three PVDF composites with different fillers as examples, we built the 3D stochastic breakdown model to study the effect of filler on electric field distribution. Here, *ε*_r_ of PVDF matrix and SiO_2_, Al_2_O_3_, and TiO_2_ fillers are fixed with the values of 10, 4, 10, 59, as shown in **Figure** **4**a–c, respectively. It can be seen that the local electric field could be distorted in the interior regions of PVDF composites owing to the large mismatch of *ε*_r_ between PVDF matrix and SiO_2_, TiO_2_ fillers. Although the *ε*_r_ of SiO_2_ is lower than of Al_2_O_3_, the mismatch of the *ε*_r_ between the matrix and the fillers leads to different degrees of electric field distortion, which ultimately affects the *E*_b_ of the polymer‐based composites. To clarify the underlying physical mechanism behind those differences, Figure 4d1–d3 illustrates a simplified polymer‐based composite model with different fillers. The development of the breakdown path generally requires a continuous cycle of matrix region – transition region, transition region – filler region, filler region – transition region, and transition region – matrix region (certainly, the filler agglomeration is not considered here). When the breakdown path spreads, no matter from the low *ε*_r_ region to the high *ε*_r_ region or from the high *ε*_r_ region to the low *ε*_r_ region, owing to without enough/excessive compensating charges, the polarized charges would build a depolarization field whose direction is opposite to the applied electric field. The local electric field is determined by the contributions from both the applied electric field and the reverse depolarization field, which leads to the local electric field distortion. The breakdown path is easier and more favorable for developing the electric field distortion, which is not conducive to the improvement of the *E*_b_ of the polymer‐based composites. This also explains why current polymer‐based composite microstructure designs favor multilayer or gradient structures (through the possibility of falling dielectric mismatch between layers).^[^ ^40^, ^41^, ^42^, ^43^ ^]^

**Figure 5:**
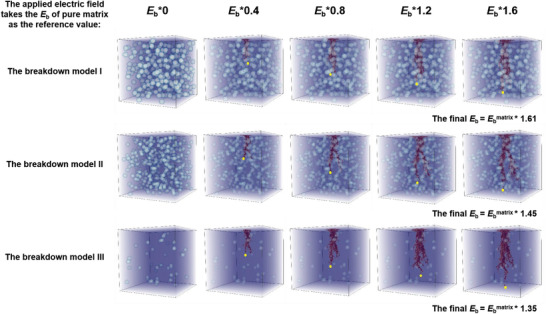
The breakdown path evolution of the PVDF composites was simulated by adjusting three variables at the same time under the change of an applied electric field.

On the basis of the abovementioned simulation results, the blocking effect, filling effect, and dielectric matching, which can be adjusted by the three variables *d*, *v*, and *ε*_r_ of filler, were found to be significant on the *E*_b_ of the polymer‐based composites. Through the high‐throughput stochastic breakdown simulation results (Figures S4–S15, Supporting Information), the breakdown path evolution of polymer‐based composites can be observed after adjusting the three variables, and a general rule can be obtained: in a specific range, the larger the *d* of the filler, the more appropriate filling state, and the closer *ε*_r_ of fillers to the matrix, the *E*_b_ of the polymer‐based composite improvement is more significant. In order to observe the physical process of internal breakdown of polymer‐based composites under the coupling effect of three variables (i.e., adjusting three variables simultaneously) in detail, three 3D stochastic breakdown models of PVDF composites were built, respectively. The breakdown model I (filled Al_2_O_3_ with 60 nm and 7 vol%), the breakdown model II (filled SiO_2_ with 10 nm and 10 vol%), and the breakdown model III (filled TiO_2_ with 10 nm and 1 vol%), the breakdown evolution process with electric field increasing is shown in **Figure** **5**. The results of directional simulation are consistent with the prediction, the breakdown model I has the highest *E*_b_, and it can be seen that the improvement of *E*_b_ becomes more complicated under the coupling effect of filler *d*, *v*, and *ε*_r_. The high‐throughput stochastic breakdown simulation database of this work will provide certain guidance for future study work.

### 2.2 Machine Learning Study of Breakdown Strength and Energy Storage

The machine learning database was established based on the *E*_b_ results of 504 groups of high‐throughput stochastic breakdown simulations, and dielectric constant *ε*_r_, size *d*, and content *v* of filler were selected as variables to build an interpretable machine learning model. Here, *ε*_r_ and *E*_b_ are normalized so that the resulting expression is suitable for wider range of polymers. 12 prototypical functions (*x*, *x* ^2^, *x* ^3^, *x* ^−1^, *x* ^−2^, *x* ^−3^, ln(*x*), ln(*x*) ^−1^, e*^x^*, e^−^ *^x^*, *x* ^1/2^, *x* ^−1/2^) and 4 interactions (*x*_1_&*x*_2_, *x*_1_&*x*_3_, *x*_2_&*x*_3_, *x*_1_&*x*_2_&*x*_3_) are considered to generate descriptors to perform the regression analysis by Least Squares Regression (LSR). The coefficient of determination (adjusted‐*R* ^2^) of the LSR is used as the criterion for screening of descriptors. Three rounds of screening are performed to obtain the final prediction expression about *E*_b_.

**Figure 6:**
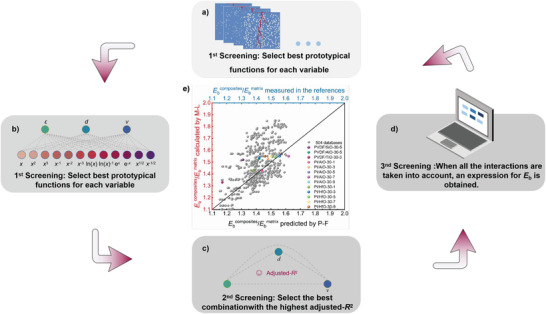
a–d) The detailed machine learning process flow on studying breakdown strength of polymer‐based composites. e) Comparisons of normalized breakdown strengths between the stochastic breakdown model and the machine learning prediction, verified by multiple experimental results (some included in the database, others not, marked as the colored solid symbols). The experimental breakdown strengths of polymer‐based composites are cited from the references.^[^ ^21^, ^39^ ^]^

A detailed workflow of the regression analysis of the *E*_b_ is shown in **Figure** **6**. As the first step, based on the stochastic breakdown model, 504 *E*_b_ values of different polymer‐based composites are calculated as the regression analysis database. Here, *Y*, *x*_1_, *x*_2_, and *x*_3_ represent *E*_b_^composites^ /*E*_b_^matrix^, *d*_filler_, *v*_filler_, and *ε*_r_^filler^/*ε*_r_^matrix^, respectively. 1st screening is performed by ranking the coefficient of determination adjusted‐*R* ^2^ by LSR to find the best combination of variables with one function. Table S2 (Supporting Information) shows the regression results of combinations of each variable with one function in 1st screening. It is found that all regression results of *d* and *v* combined with only one prototypical function exhibit low adjusted‐*R* ^2^, of which *x* ^2^ function and e^−^ *^x^* function can lend to better adjusted‐*R* ^2^ = 0.007 and adjusted‐*R* ^2^ = 0.009 for size *d* and content *v* than other functions. What's more, the *ε*_r_ shows a best adjusted‐*R* ^2^ = 0.743 with *x* ^−1/2^ function, the *E*_b_ is more dependent on *ε*_r_. It is expected that the *ε*_r_ plays a decisive role.

Based on the 1st round of screening, since *ε*_r_ has a greater influence on *E*_b_, the top three functions for *x*_1_ variable are selected, together with the best functions of *x*_2_ and *x*_3_, to compose the compound descriptors with two and three dependent variables in 2nd round regression and screening. The regression results are shown in Table S3 (Supporting Information). For two variables, *ε*_r_ with ln(*x*) function and *d* with *x* ^2^ function can result in a better adjusted‐*R* ^2^ = 0.34, *ε*_r_ with ln(*x*) function and *v* with e^−^ *^x^* function can result in a better adjusted‐*R* ^2^ = 0.09, *d* with *x* ^2^ function and *v* with e^−^ *^x^* function are not considered because their adjusted‐*R* ^2^ is low when they are considered independently. Furthermore, three variables with three best prototypical functions are performed to find the best predictive expression. As shown in Table S3 (Supporting Information), *ε*_r_ & *d* & *v* with orderly ln(*x*) & *x* ^2^ & e^−^ *^x^* functions exhibit the best adjusted‐*R* ^2^ = 0.042. Next, the interactions among variables with corresponding best prototypical function are considered based on the results of the 2nd round of regressions, and the results are shown in Table S4 (Supporting Information). When all interactions are taken into account, the best adjusted‐*R* ^2^ = 0.778 can be obtained. Since the effects of *ε*_r_, *d*, and *v* on *E*_b_ are complex, this is an acceptable result. In addition, it can be seen from Table S4 (Supporting Information) that inspection significance (*P*) serves as the analysis standard to inspection the correlation between each term and whole of the expression. For *x*_1_, when other variables remain unchanged, its influence on the dependent variable is statistically significant, and the partial regression coefficient is less than 0, indicating a negative correlation with the dependent variable *Y*; for *x*_2_, when other variables remain unchanged, its influence on the dependent variable is statistically significant, and the partial regression coefficient is greater than 0, indicating a positive correlation with the dependent variable *Y*; for *x*_3_, when other variables remain unchanged, its influence on the dependent variable is statistically significant, and the partial regression coefficient is less than 0, indicating a negative correlation with the dependent variable *Y*; for *x*_1_ & *x*_2_, when other variables remain unchanged, its influence on the dependent variable is statistically significant, and the partial regression coefficient is greater than 0, indicating a positive correlation with the dependent variable *Y*; for *x*_1_ & *x*_3_, when other variables remain unchanged, its influence on the dependent variable is statistically significant, and the partial regression coefficient is less than 0, indicating a negative correlation with the dependent variable *Y*; however, for *x*_1_ & *x*_2_ & *x*_3_, it indicates that its influence on the dependent variable has no statistical significance when other variables remain unchanged, because the *P* value is greater than 0.05. Therefore, the expression given by

(1) $$\begin{array}{ccc} Y & = & 1.455-0.089{\left(x_{1}\right)}^{-1/2}+0.00012{\left(x_{2}\right)}^{2}-0156e^{-(x_{3})}\\ & & +\;0.000006\;\ln\left(x_{1}\right){\left(x_{2}\right)}^{2}-0.061\;\ln\left(x_{1}\right)e^{-(x_{3})} \end{array}$$

Figure 6 also shows the comparisons of *E*_b_ calculated from the stochastic breakdown simulations and predicted from the machine learning model. It is seen that most of the database (using gray symbols) is clustered near the black straight line, indicating that most of the prediction results are in good agreement with the stochastic breakdown simulation results. Certainly, some of the data are above the black straight line, after all, considering the coupling effect of various variables on *E*_b_, these jumping points are acceptable, which also corresponds to the adjusted‐*R*_2_ = 0.778 of regression analysis result, which is close to 1. In addition, additional literature results were used to verify the machine learning results (used as the colored symbols), and the polymer‐based composites parameters used in the additional machine learning are listed in Table S5 (Supporting Information).^[^ ^21^, ^39^ ^]^ The majority of the data points are located around the black straight line, which indicates that our machine learning can be treated as a predictive model. It should be noted that some of these data points were used for high‐throughput stochastic breakdown simulation and machine learning, while others were outside the database. The reason for selecting data points in this way is not only to verify the accuracy of the model, but also to verify its universality.

Due to the complex breakdown mechanism inside polymer‐based composites, there are not many attempts to predict the *E*_b_, but many attempts to predict the *ε*_r_.^[^ ^44^ ^]^ The polarization properties of polymer‐based composites are a function of the filler's geometries and orientations relative to an applied electric field. Three depolarization factors (*N_x_*, *N_y_*, *N_z_*) describe the degree of reduced polarization in the fillers.^[^ ^45^ ^]^ The depolarization factor can be expressed by integrating the following equation

(2) $$N_{x}=\frac{a_{x}a_{y}a_{z}}{2}\int_{0}^{\infty }\frac{1}{\left(s+a_{x}^{2}\right)\sqrt{\left(s+a_{x}^{2}\right)\left(s+a_{y}^{2}\right)\left(s+a_{z}^{2}\right)}}ds$$

where *a_x_*, *a_y_*, and *a_z_* represents the three axes in the *x*, *y*, and *z* directions, respectively. For spherical fillers, the three depolarization factors (*N_x_*, *N_y_*, *N_z_*) are (1/3, 1/3, 1/3), so the *ε*_r_ of polymer‐based composites can be calculated by Maxwell–Garnett (MG) model^[^ ^46^, ^47^ ^]^

(3) $$\varepsilon_{\mathrm{eff}}=\varepsilon_{M}\left[\frac{\varepsilon_{F}+2\varepsilon_{M}+2v_{F}\left(\varepsilon_{F}-\varepsilon_{M}\right)}{\varepsilon_{F}+2\varepsilon_{M}-v_{F}\left(\varepsilon_{F}-\varepsilon_{M}\right)}\right]$$

where *ε*_F_ is the dielectric constant of the filler, *ε*_M_ is the dielectric constant of the matrix, and *v*_F_ is the volume fraction of the fillers. According to the calculation formula of *U* mentioned in the introduction, the *ε*_r_ prediction expression and *E*_b_ prediction expression are substituted into the *U* calculation formula, and get the final prediction expression about *U*

(4) $$\begin{array}{ccc} U & = & \frac{1}{2}\varepsilon_{0}\varepsilon_{M}\left[\frac{\varepsilon_{F}+2\varepsilon_{M}+2v_{F}\left(\varepsilon_{F}-\varepsilon_{M}\right)}{\varepsilon_{F}+2\varepsilon_{M}-v_{F}\left(\varepsilon_{F}-\varepsilon_{M}\right)}\right]\\ & & \;\;\;\;\;\;\;\;\;\;\times \;{\left\{\left[\begin{array}{c} 1.455-0.089{\left(\varepsilon_{F}\right)}^{-1/2}+0.00012{\left(d_{F}\right)}^{2}-0.156e^{-\left(v_{F}\right)}\\ +0.000006\ln\left(\varepsilon_{F}\right){\left(d_{F}\right)}^{2}-0.061\ln\left(\varepsilon_{F}\right)e^{-\left(v_{F}\right)} \end{array}\right]E_{b}^{M}\right\}}^{2} \end{array}$$

where *ε*_0_ is the vacuum dielectric constant, generally ≈ 8.85×10^−12^ F m^−1^, *E*_b_^M^ is the breakdown strength of the matrix. In this expression, only some parameters of matrix and fillers can be used to calculate the *U*, which is suitable for different polymers.

### 2.3 Microstructure Characterization of Directional Experiments

**Figure 7:**
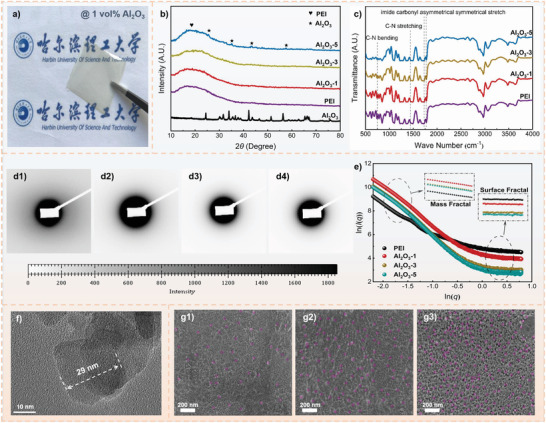
a) The PEI/Al_2_O_3_ composites; b) XRD of Al_2_O_3_, PEI, and the PEI/Al_2_O_3_ composites; c) FTIR of PEI and PEI/Al_2_O_3_ composites; d) the original 2D SAXS patterns of the polymer‐based composites. e) Logarithmic curves of PEI/Al_2_O_3_ composites. f) The TEM image of Al_2_O_3_ fillers. g) The SEM images of the PEI/Al_2_O_3_ composites.

In this section, we present the directional experiment results in verifying the correctness of stochastic breakdown simulation results and machine learning predictions, and we choose PEI/Al_2_O_3_ composites as the target material. More details about the experimental preparation are discussed in the Supporting Information. The PEI/Al_2_O_3_ composites were systematically characterized, and the details of the characterization were shown in **Figure** **7**. Figure 7a shows the morphology of the PEI/Al_2_O_3_ composites, it can be seen that the sizable surface of the transparent film has no defects. As illustrated in Figure 7b of the XRD patterns for Al_2_O_3_ fillers, pure PEI and the PEI/Al_2_O_3_ composites with different *v* fillers (1, 3, and 5 vol%), the characteristic diffraction peaks of PEI matrix and Al_2_O_3_ are indexed and labeled. For PEI, there is only one diffusion scattering peak located at about 20°. For the PEI/Al_2_O_3_ composites, the peak belonging to the PEI is also found at about 20° (heart‐shaped mark), indicating that the introduction of Al_2_O_3_ did not damage the crystal structure of PEI matrix itself. The characteristic diffraction peaks for Al_2_O_3_ are at 2*θ* ≈24.5°, ≈32.2°, ≈35.6°, ≈43.1°, and ≈55.9°, respectively (star‐shaped mark). It is worth noting that the barely visible characteristic diffraction peaks of Al_2_O_3_ can be detected in the PEI/Al_2_O_3_‐5 vol% composite, nevertheless the peaks are too weak in the PEI/Al_2_O_3_‐1 and 3 vol% composites, due to the small volume fraction of Al_2_O_3_ inside the probed volume of the composites, which dominated by the high scattering strength of the PEI matrix itself. As shown in Figure 7c of the FTIR spectra of pure PEI and the PEI/Al_2_O_3_ composites, the characteristic absorption peaks of PEI are distributed around 750, 1380, and 1780 cm^−1^. The peaks located at 750 and 1380 cm^−1^ correspond to the C—N bending and C—N stretching. The peaks located at 1720 and 1780 cm^−1^ belong to the asymmetric symmetric stretching of the imide carbonyl group in the PEI matrix.^[^ ^48^ ^]^

Small angle X‐ray scattering (SAXS) has been proven to be effective in detecting the microstructure of polymer‐based composites,^[^ ^49^, ^50^, ^51^ ^]^ SAXS is used in this study to investigate the microstructure of PEI/Al_2_O_3_ composites, and the original 2D SAXS patterns of the polymer‐based composites are shown in Figure 7d1–d4. The fractal dimension parameter *D* is used to quantify mass or surface changes of scatters in the polymer‐based composites. The fractal characteristics of multiphase composites can be divided into mass fractal (*D*_m_) and surface fractal (*D*_s_). *D*_m_ reflects the mass distribution in the polymer‐based composite, and *D*_s_ reflects the surface roughness of the polymer‐based composite. Figure 7e is the log–log curve of the PEI/Al_2_O_3_ composites, the coexistence of *D*_m_ and *D*_s_ can be clearly identified. It indicates that the distribution and aggregation of Al_2_O_3_ are random nonlinear processes, which is also consistent with the establishment of the stochastic breakdown model, and that all the polymer‐based composites are relatively compact in structure. In addition, it was found that *D*_m_ and *D*_s_ of the PEI composites did not change with the increase of *v* of Al_2_O_3_ in the plots in Figure 7e, indicating that all the composites had excellent organic–inorganic compatibility. Additional SAXS analysis can be found in the Supporting Information. In order to confirm the *d* of fillers and ensure the accuracy of the subsequent stochastic breakdown simulations, the TEM image of Al_2_O_3_ is shown in Figure 7f. It can be observed and determined that the *d* of Al_2_O_3_ particle is about 29 nm. The cross‐section SEM images of three polymer‐based composites are shown in Figure 7g1–g3, the SEM can be used to observe the microstructure of polymer‐based composites more intuitively. Combined with TEM image, it can be seen that Al_2_O_3_ is well dispersed, and the amount of Al_2_O_3_ increases significantly with the increase of *v*. All the characterization results showed that the PEI/Al_2_O_3_ composites were successfully synthesized and Al_2_O_3_ was uniformly dispersed without agglomeration, basically conforming to the fillers distribution characteristics in the stochastic breakdown model.

### 2.4 Electrical Performance Test of Directional Experiments

**Figure 8:**
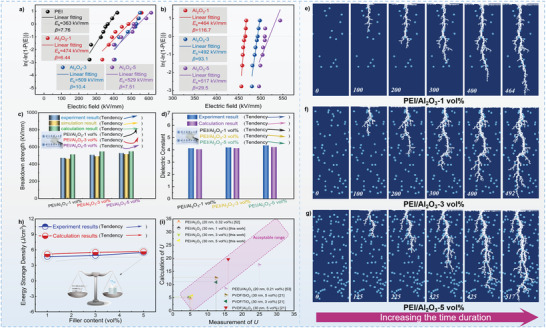
a) The experimental dielectric breakdown strengths of a PEI and the PEI/Al_2_O_3_ composites; b) The simulated dielectric breakdown strengths of PEI/Al_2_O_3_ composites. c) Dielectric breakdown strength comparison of directional experiments, stochastic breakdown simulation and machine learning results of PEI and PEI/Al_2_O_3_ composites. d) Dielectric constant comparison of directional experiment and calculation results. e–g) The stochastic breakdown simulation of PEI/Al_2_O_3_ composites. h) Energy storage density comparison of experiment results and calculation results in this work. i) Energy storage density comparison of directional experimental and calculation results from the published works.^[^ ^21^, ^52^, ^53^ ^]^

The two‐parameter Weibull statistical distribution function was used to accumulate the characteristic breakdown strength based on the cumulative breakdown probability, and the equation can be described as follows

(5) $$\ln\left(-\ln\left(1-P\left(E\right)\right)\right)=\beta \ln\left(E\right)-\beta \ln\left(E_{b}\right)$$

where *P*(*E*) is the cumulative breakdown probability, *E* is the experimental measured breakdown strength of the composites, *E*_b_ is the breakdown strength at *P* = 63.2%, and the shape parameter *β* evaluates the scatter of the breakdown data collected in experiment. The measured *E*_b_ of the pure PEI and PEI/Al_2_O_3_ composites were plotted in a two‐parameter Weibull statistical distribution function as shown in **Figure** **8**a, with *E*_b_ of 363, 474, 509, and 529 kV mm^−1^ for pure PEI and the PEI/Al_2_O_3_ composites with 1, 3, and 5 vol% Al_2_O_3_, respectively. As expected, Al_2_O_3_ has better electric field endurance than the PEI matrix. Consequently, Al_2_O_3_ can partake and assimilate the major portion of the voltage in the PEI/Al_2_O_3_ composites, leading to an enhanced *E*_b_ of polymer‐based composites. The stochastic breakdown simulation of PEI/Al_2_O_3_ composites was carried out, and the results are shown in Figure S19 (Supporting Information). In each stochastic breakdown simulation process, the random distribution of fillers would have some influence on the breakdown path and *E*_b_, so the stochastic breakdown simulations were repeated 8 times for each PEI/Al_2_O_3_ composite, and the simulation results can be more accurate by using the two‐parameter Weibull statistical distribution function. The simulated *E*_b_ of the PEI/Al_2_O_3_ composites is shown in Figure 8b, with *E*_b_ of 464, 492, and 517 kV mm^−1^ for the PEI/Al_2_O_3_ composites with 1, 3, and 5 vol% Al_2_O_3_, respectively. The simulated results are in agreement with the experimental results within less than 3.5%, within the acceptable range. Both the results of experimental test and the stochastic breakdown simulation show that the improvement of *E*_b_ of PEI/Al_2_O_3_ composites by increasing Al_2_O_3_ content is consistent. Figure 8e–g shows that the development and evolution of the breakdown paths of PEI composites, from which the influence of filling effects on the breakdown path can be clearly observed. Because Al_2_O_3_ is a better insulator, the insulation properties become stronger as the *v* increases, but when the *v* exceeds a certain critical value, the agglomeration effect occurs in the polymer‐based composites, which is inevitable. To further test the reliability of the *E*_b_ function, Figure 8c shows a comparison of experimentally measured, the stochastic breakdown simulated, and machine learning calculated *E*_b_ values, the figure clearly shows the variation trends of *E*_b_ under various conditions. The trends of the experiment, the simulation and the calculation results are consistent. With the increase of *v* of fillers, the *E*_b_ increases. However, because the calculation expression is universal and a high *E*_b_ is obtained even in a small amount of filling, the increasing trend is slow.

The accuracy of the *ε_r_* prediction is verified by the dielectric tests. By the MG model calculation, *ε*_r_^(PEI/Al2O3‐1 vol%)^ = 4.05, *ε*_r_^(PEI/Al2O3‐3 vol%)^ = 4.14, *ε*_r_^(PEI/Al2O3‐5 vol%)^ = 4.23, as shown in Figure 8d, which is consistent with experimental results and verifies the accuracy of MG model, the dielectric performance test results can be found in Figure S20 (Supporting Information). Despite the *ε*_r_ of all polymer‐based composites has been increased slightly, some changes can still be observed, which are mainly attributed to the following reasons. First, the *ε*_r_ of Al_2_O_3_ is higher than that of PEI, and its introduction can improve the *ε*_r_ of polymer‐based composites. Meanwhile, the introduction of a large number of inorganic fillers into the polymer increases the possibility of interfacial polarization. Finally, based on the results of dielectric and breakdown experiments, the *U* of PEI/Al_2_O_3_ composites were calculated by using the general energy storage formula. As shown in Figure 8h, it is seen that the *U* reaches ≈4.7, 4.9, and 5.5 J cm^−3^, when the *v* of Al_2_O_3_ is 1, 3, and 5 vol%, respectively. As the filler content increases, the *U* of polymer‐based composite increases. The increase in *U* is well understood because *ε*_r_ and *E*_b_ of polymer‐based composites are optimized with the introduction of Al_2_O_3_. At the same time, the *U* of PEI/Al_2_O_3_ composites are calculated by using the predictive expression obtained in this work, the prediction *U* reaches ≈5.1, 5.5, and 5.6 J cm^−3^, when the *v* of Al_2_O_3_ is 1, 3, and 5 vol%, respectively. Figure 8h also shows the *U* comparison of the experimental and the calculation results, the calculation results (the red line) are consistent with the experiment results (the blue line), with the increases of *v*, the *U* of polymer‐based composites is increased, and it can be seen that the *U* values from the experiment and the simulation are quite close, which also confirms the feasibility and accuracy of this work. There is slight bigger gaps in the low loading part, and we believe that because the main contributor to *U* is *E*_b_ (*U* is proportional to the quadratic of *E*_b_), while the *E*_b_ expression predicts slightly higher *E*_b_ with a small amount of *v*_F_. In addition, some works with nanoparticle filled polymer‐based composites were searched and compiled, and the predicted *U* were obtained by calculating the parameters through expression we derived.^[^ ^21^, ^52^, ^53^ ^]^ The predict *U* values compared with the measured *U* values as shown in Figure 8i. It can be seen that the predicted values and the measured values are close to follow 45° line, within some unaccounted error range, mainly due to the experimental uncertainties and the theoretical approximation of the formulation. The *U* prediction expression of this work is intended to provide some general for researchers in the field to screen fillers and polymer‐based composites microstructure designs.

## 3 Conclusion

In summary, through high‐throughput stochastic breakdown simulation and machine learning methods, the *E*_b_ of two typical polymer‐based composites under multiple variables was studied. Established a model to explore the influential mechanisms of each variable on *E*_b_. It is found that the blocking effect, the filling effect and the dielectric mismatch have strong impact on the *E*_b_ of the polymer‐based composites. The *E*_b_ expression with high fitting degree was obtained by multiple linear regression equations. Combined with the classical dielectric calculation equation, the *U* expression suitable for most polymers was finally obtained. Comprehensive directional experiments of microstructure characterization were also carried out. The stochastic breakdown model and the prediction expression are verified by the directional experiments. The experiment results, simulation results and calculation results are all in agreement, which indicates the universality and accuracy of the prediction expression in this work. This work provides convenience and guidance for researchers in developing polymer dielectric capacitors with high energy storage density.

## 4 Experimental Section

### Stochastic Breakdown Model

A modified stochastic model (commercial software, MuPRO dielectric breakdown module) was employed based on the one proposed by Niemeyer et al. to study the internal electric field distribution and breakdown path evolution of the polymer composites under the applied electric field.^[^ ^31^ ^]^ In the stochastic breakdown model, the breakdown probability *P*(r) is written as

(6) $$P\left(r\right)=\frac{E{\left(r\right)}^{2}}{E_{b}{\left(r\right)}^{2}}/\sum \frac{E{\left(r\right)}^{2}}{E_{b}{\left(r\right)}^{2}}$$

where *E*(r) is the electric field of a local point determined by the externally applied voltage and the microstructure, and *E*_b_(*r*) is the corresponding intrinsic breakdown strength determined by the polymer‐based composites, and the summation in the denominator is the sum over all points that the local electric field exceeds the breakdown strength.

The local electric field distribution is obtained by solving the electric equilibrium equation using an spectral iterative perturbation method.^[^ ^30^ ^]^ More details of the stochastic breakdown model can be found in the Supporting Information.

### Machine Learning Approach

Machine learning uses multiple linear regression equations for data analysis. The breakdown strength of polymer‐based composites was taken as the dependent variable *Y*. Three parameters of the fillers, including the dielectric constant, size, content, and the new variables after the conversion of their functional forms are selected as the independent variable *x*. The Least Squares Regression (LSR) was used to fit the curves of *Y* and *x*, and the linear relationship test and the fitting degree test (adjusted‐*R* ^2^) were used to test the fitting condition of the equation. Moreover, the quadratic and cubic interaction terms of independent variable *x* were established, and the combination of independent variables with the highest fitting degree in each round was substituted into the final fitting equation. After the model was successfully built, the database was backdated for detection and prediction. In the machine learning approach in this work, inspection significance (*P*) < 0.05 is considered to be statistically significant, and the two‐way verification was used.

### Experimental Verification

Materials, synthesize methods, and characterization methods for directional experiments can be found in the accompanied Supporting Information. Electrical property testing information of the PEI/Al_2_O_3_ composites is as following. The Al electrodes (25 and 3 mm in diameter) were evaporated on two sides of the PEI/Al_2_O_3_ composites for the following electric measurements. The dielectric constants of the composites were measured by Concept 40 wide‐band dielectric spectrometer at room temperature from 1 to 10^7^ Hz. The breakdown strength was investigated by an electric breakdown tester (YDZ‐560) in silicone oil at a voltage ramp of 500 V s^−1^. The test electrode for the breakdown strength was a ball‐plate electrode, 8 data points were collected for each sample, and the two‐parameter Weibull statistics distribution function was carried out.

## Conflict of Interest

The authors declare no conflict of interest.

## Supporting information

Supporting information

## Data Availability

Research data are not shared.
