# Supplementary material for: Prediction of Energy Storage Performance in Polymer Composites Using High‐Throughput Stochastic Breakdown Simulation and Machine Learning
Source: Adv Sci (Weinh). 2022 Apr 10;9(17):2105773. doi: 10.1002/advs.202105773 (PMC9189649; doi:10.1002/advs.202105773)
Supplement: Supplementary file 1 — Supporting information [file ADVS-9-2105773-s001.pdf]

ADVANCED SCIENCE
Open Access

Supporting Information

for *Adv. Sci.*, DOI 10.1002/advs.202105773

Prediction of Energy Storage Performance in Polymer Composites Using High-Throughput Stochastic Breakdown Simulation and Machine Learning

Dong Yue, Yu Feng, Xiao-Xu Liu, Jing-Hua Yin*, Wen-Chao Zhang, Hai Guo, Bo Su and Qing-Quan Lei*

Supporting Information

Prediction of energy storage performance in polymer composites using high-throughput stochastic breakdown simulation and machine learning

Dong Yue, Yu Feng, Xiao-Xu Liu, Jing-Hua Yin,* Wen-Chao Zhang, Hai Guo, Bo Su, and Qing-Quan Lei*

Schematic of Energy Storage

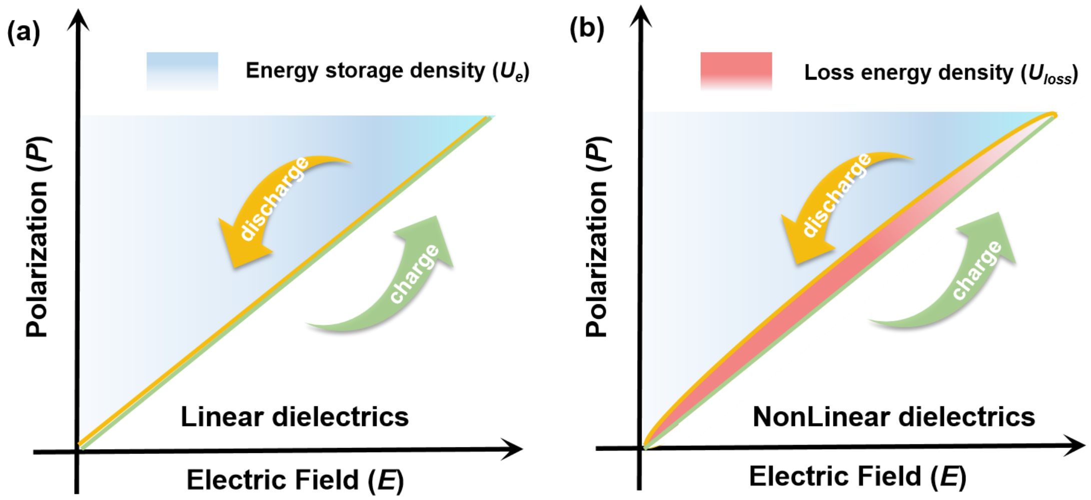

Figure S1. Schematic diagram of energy storage of (a) linear dielectrics; and (b) nonlinear dielectrics.

Stochastic breakdown Model

In the stochastic breakdown model, the breakdown probability P(r) is written as

$$P(r) = \frac{E(r)^2}{E_b(r)^2} \Big/ \sum \frac{E(r)^2}{E_b(r)^2} \tag{1}$$

where E(r) is the electric field of a local point determined by the externally applied voltage and the microstructure, and E_b(r) is the corresponding intrinsic breakdown strength determined by the polymer-based composites, and the summation in the denominator is the sum over all points that the local electric field exceeds the breakdown strength.

The local electric field distribution is obtained by solving the electrostatic equilibrium equation using an spectral iterative perturbation method.[1] As we assume there exist no spontaneous polarization in the system and the relative permittivity is independent of the applied field strength, the electric displacement D(r,E) can be expressed using Einstein notation as

$$D_i(r,E) = \varepsilon_0 k_{ij}(r) E_j(r) \tag{2}$$

where k(r) is the relative dielectric constant of the composite material.

According to Gauss's law the gradient of the electric displacement equals the position-dependent free charge density, i.e.

$$\frac{\partial D_i(r,E)}{\partial x_i} = \frac{\partial \left[\varepsilon_0 k_{ij}(r) E_j(r)\right]}{\partial x_i} = \rho_f(r) \tag{3}$$

We know that total electric field equals to external field, E^{ext}, plus the depolarization field E^d,

$$E_j^d(r) = -\frac{\partial \varphi(r)}{\partial x_j} \tag{4}$$

where φ(r) is the "depolarization potential". Using this substitution, Eq. 3 becomes

$$\frac{\partial \left[\varepsilon_0 k_{ij}(r)(E_j^{ext} - \frac{\partial \varphi(r)}{\partial x_j}) \right]}{\partial x_i} = \rho_f(r) \tag{5}$$

We can express the inhomogeneous relative dielectric constant as the sum of the homogeneous reference k^0_{ij} and a perturbation Δk_{ij}(r), i.e.

$$k_{ij}(r) = k_{ij}^{0} + \Delta k_{ij}(r) \tag{6}$$

After substituting Eq. 6 into Eq. 5 and rearranging we obtain

$$k_{ij}^{0} \frac{\partial^2 \varphi(r)}{\partial x_i \partial x_j} = \frac{\partial}{\partial x_i}\left[\Delta k_{ij}(r)(E_j^{ext} - \frac{\partial \varphi(r)}{\partial x_j})\right] - \frac{\rho_f(r)}{\varepsilon_0} \tag{7}$$

From which we can solve for the depolarization potential and thus the electric field. Details of the numerical method for solving this equation are described.[1]

A grid size of $N_x\Delta x \times N_y\Delta x \times N_z\Delta x$ is employed in all simulations. For all 2D simulations, $N_x = N_y = 256$, $N_z = 1$, and for all 3D simulations $N_x = N_y = N_z = 128$. As an example, the PI/Al_2O_3 composites is considered with isotropic relative permittivity of $\varepsilon^F = 10$ and $\varepsilon^M = 3.5$ for Al_2O_3 and PI, respectively. The relative permittivity of the breakdown phase ε^B is considered to be isotropic and to have a value of 10^4 to reflect its strong polarized ability due to the abundant space charge in the breakdown region. The breakdown strength of PI is 280 kV/mm through testing. Due to the lack of experimental data on the intrinsic breakdown strength of Al_2O_3 filler, a value of 800 kV/mm is assigned for it. For each polymer-based composite, all fillers are generated with random distribution without overlapping.

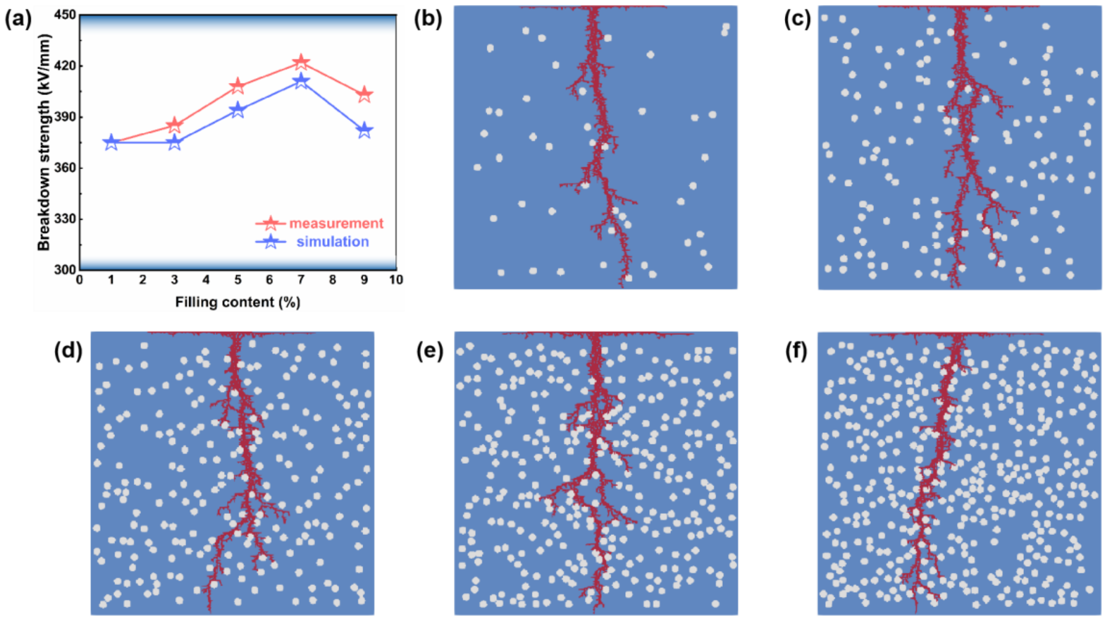

Figure S2. (a) Comparison of stochastic breakdown simulation and reference;[2] Stochastic breakdown simulation of PI/Al_2O_3 (50 nm) composite with (b)1 vol%; (c) 3 vol%; (d) 5 vol%; (e) 7 vol%; (f) 9 vol%.

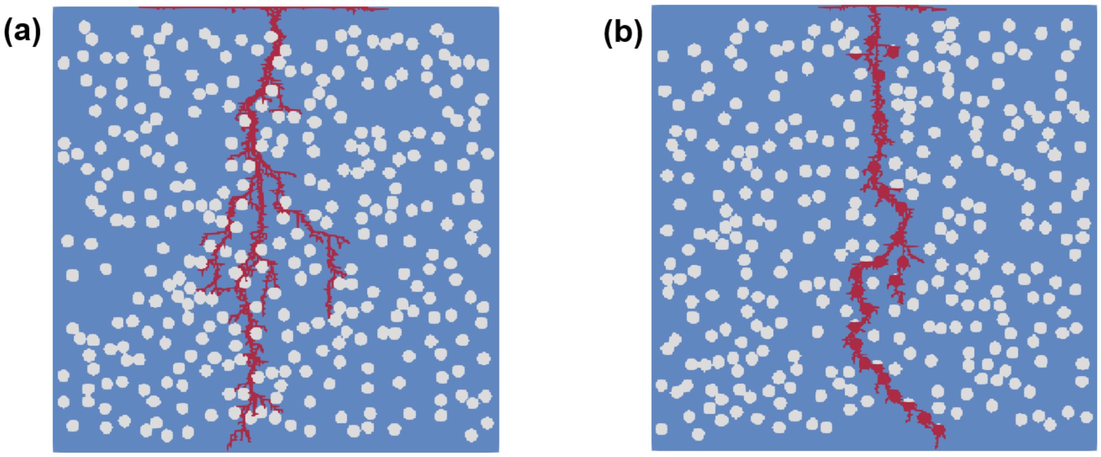

Figure S3. 2D stochastic breakdown simulations of (a) PI/SiO_2 composites and (b) PI/$BaTiO_3$ composites.

Table S1. The composite parameters used in the high-throughput simulation of this work.

Filler	Dielectric constant	Breakdown strength (kV/mm)
SiO_2	4.0	950.0
ZnO	7.0	900.0
AlN	8.5	850.0
Al_2O_3	10.0	800.0
ZrO_2	33.0	700.0
TiO_2	59.0	350.0
$BaTiO_3$	900.0	50.0

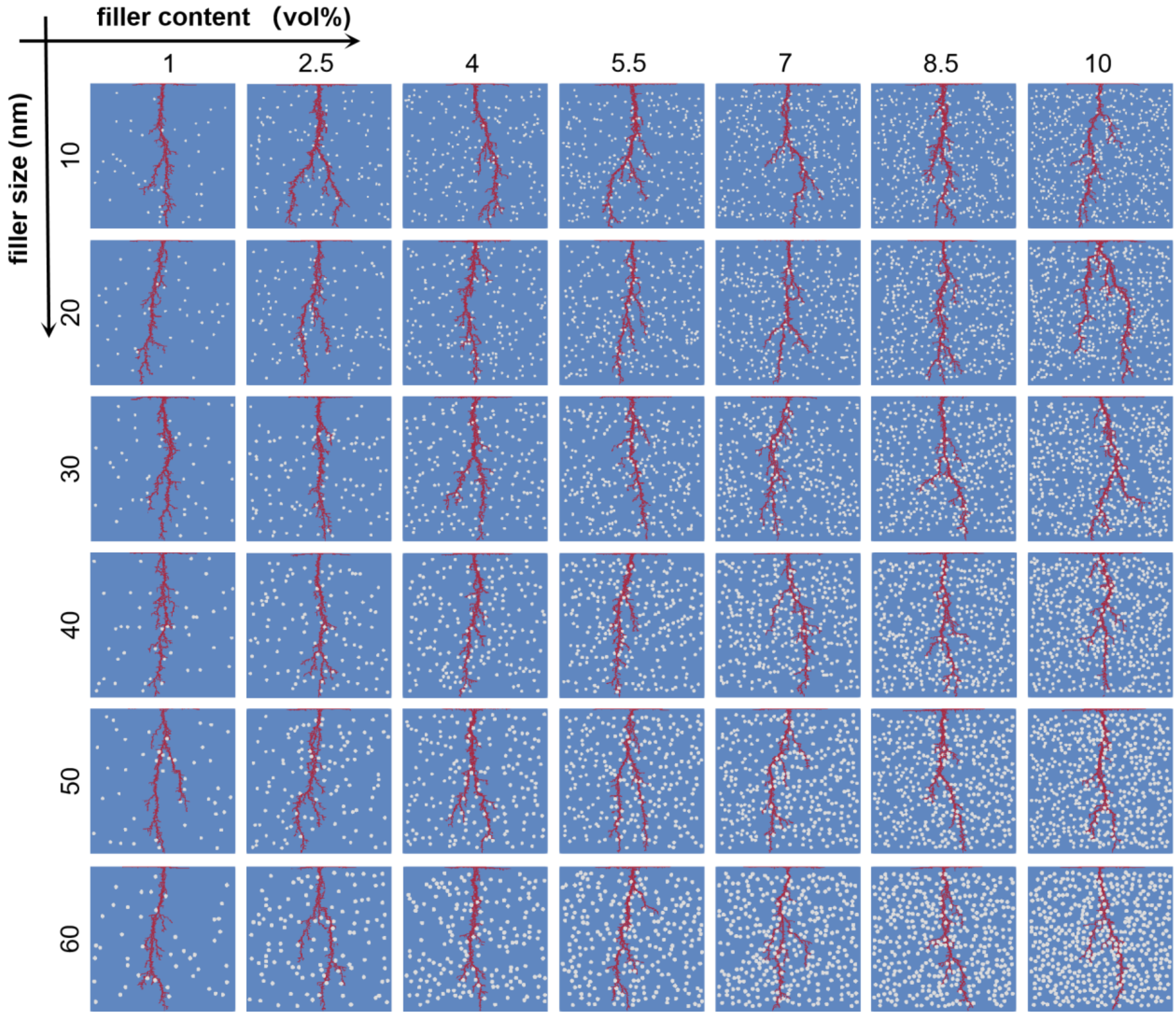

Figure S4. High-throughput stochastic breakdown simulations of PI/SiO_2 composites with different filler size and filler content.

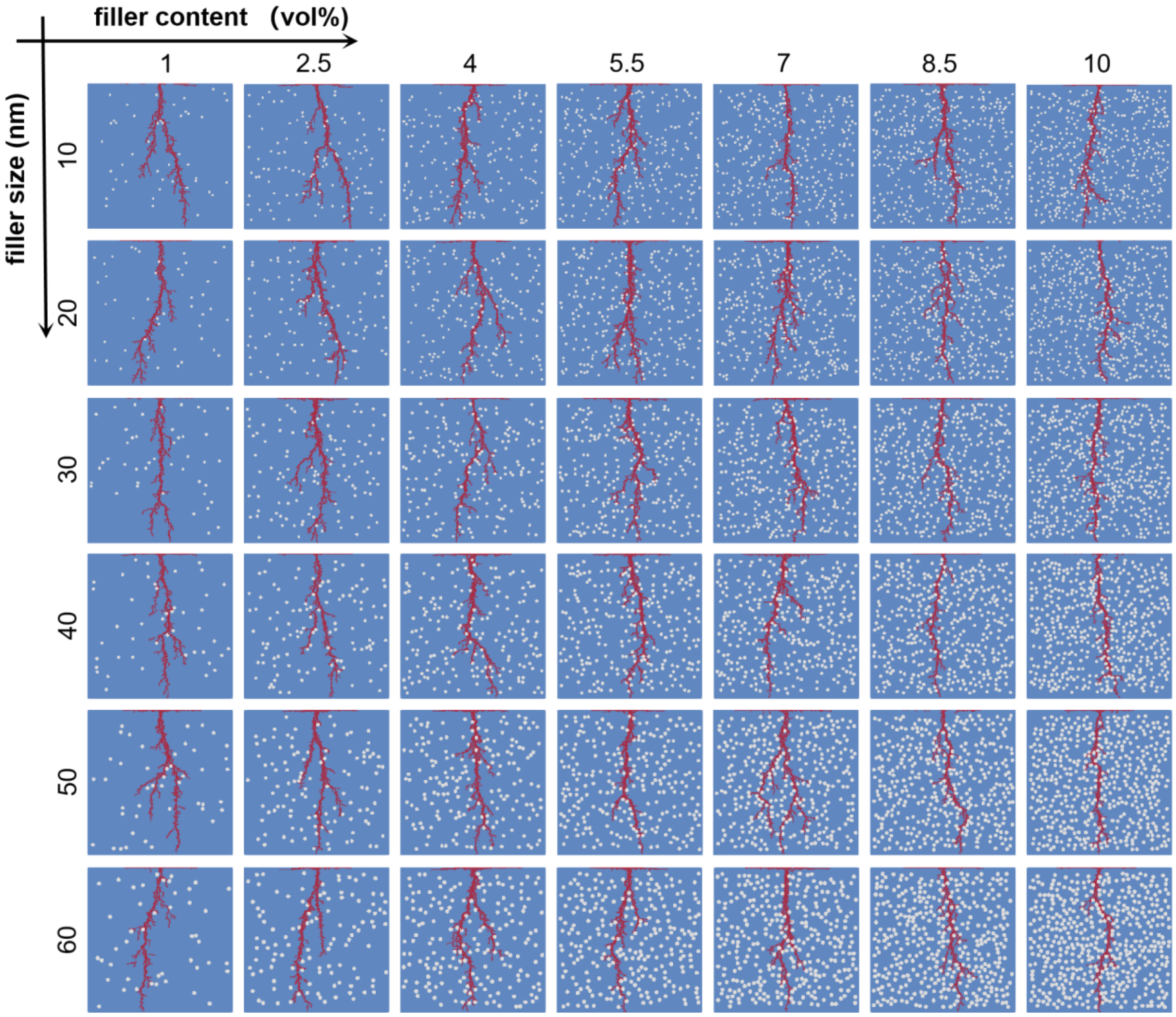

Figure S5. High-throughput stochastic breakdown simulation of PI/ZnO composites with different filler size and filler content.

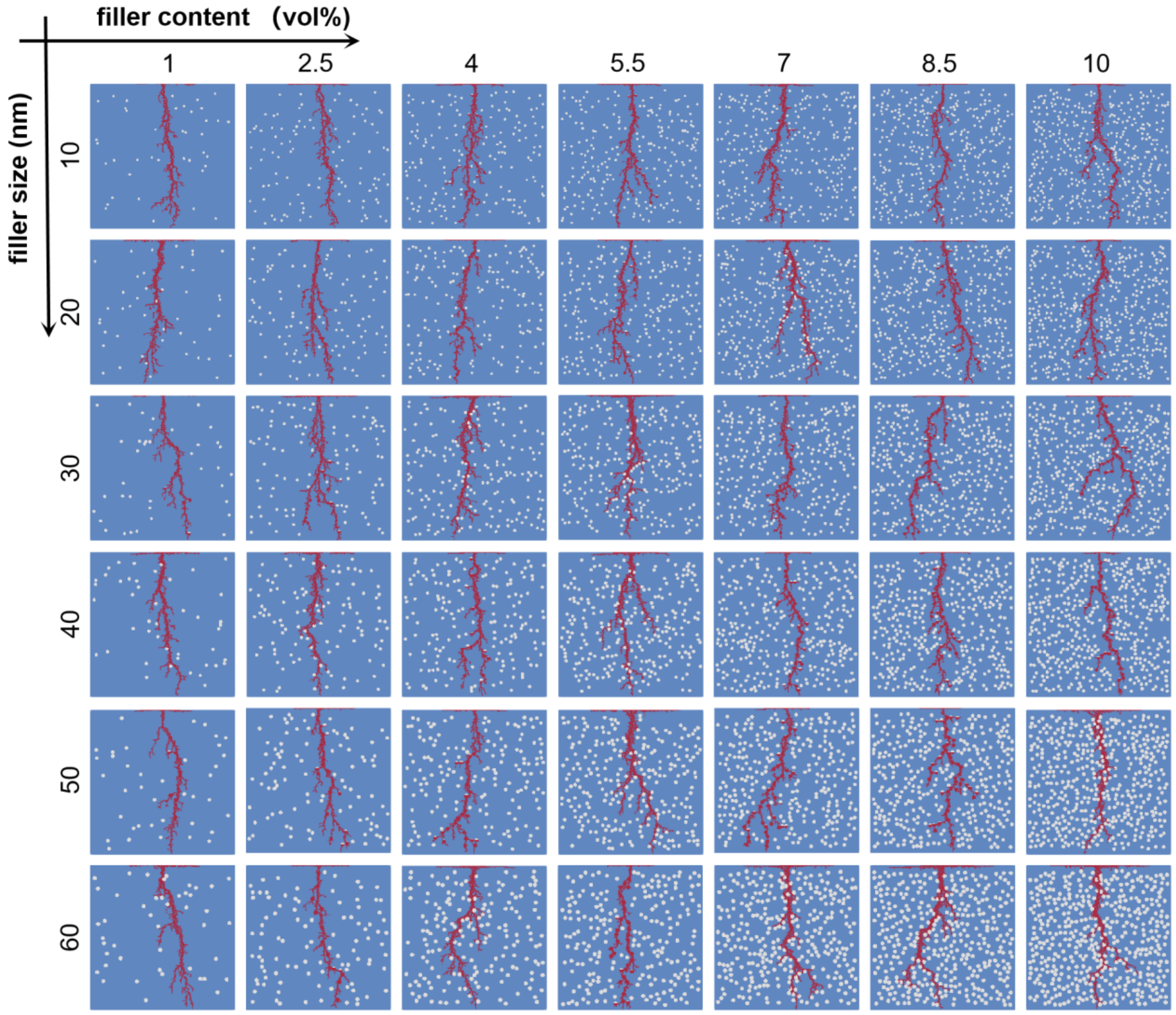

Figure S6. High-throughput stochastic breakdown simulation of PI/AlN composites with different filler size and filler content.

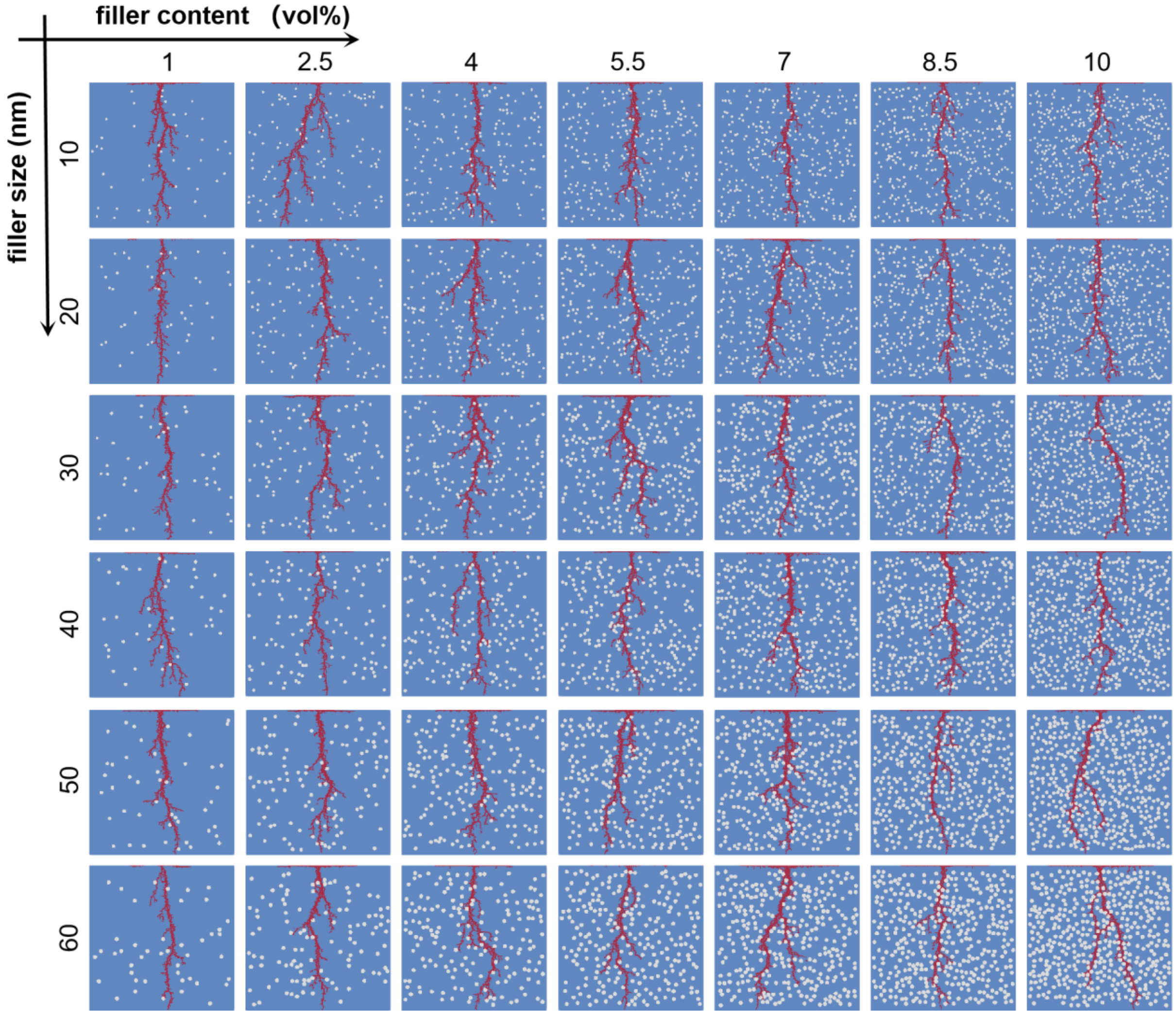

Figure S7. High-throughput stochastic breakdown simulation of PI/Al_2O_3 composites with different filler size and filler content.

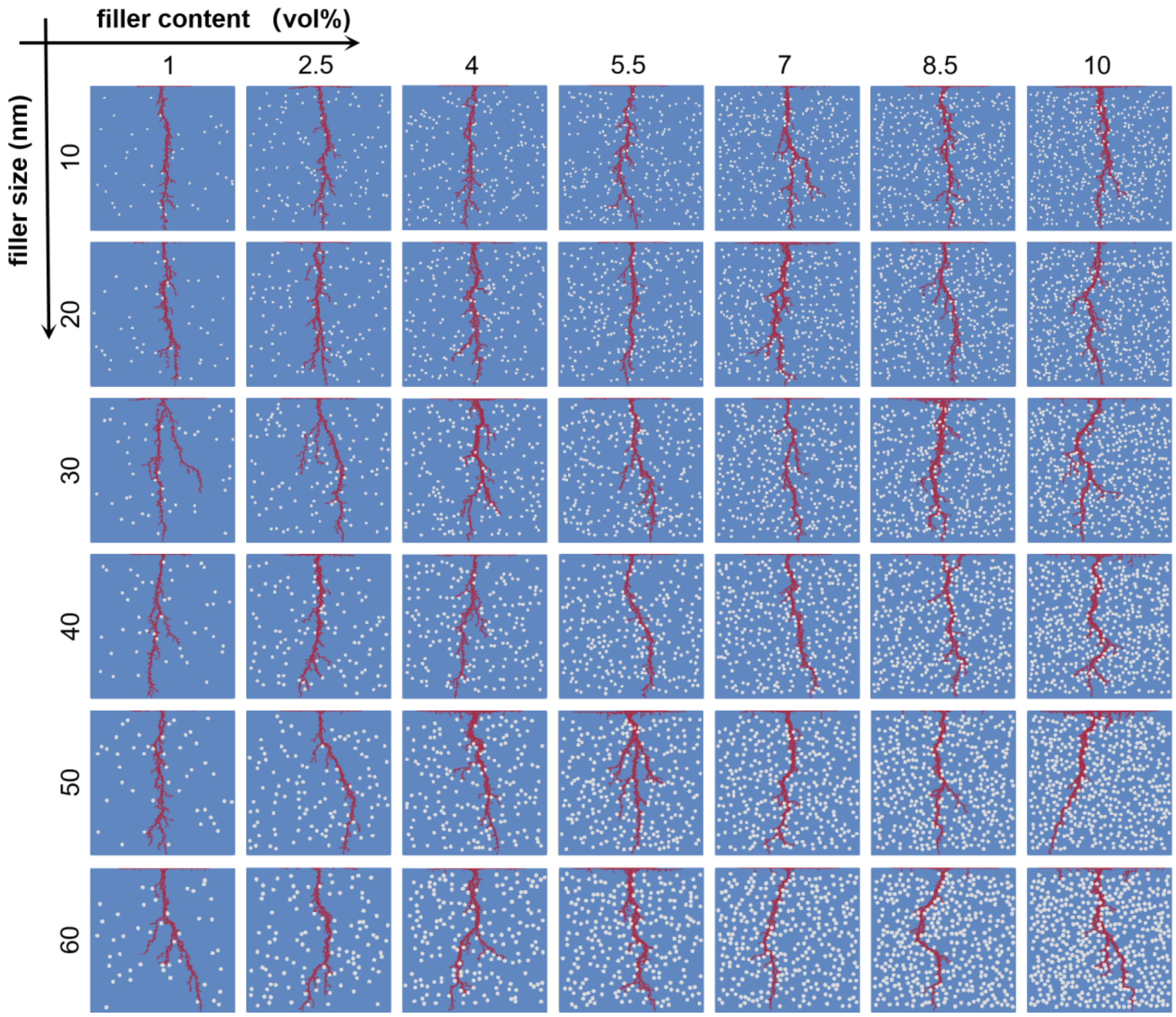

Figure S8. High-throughput stochastic breakdown simulation of PI/ZrO_2 composites with different filler size and filler content.

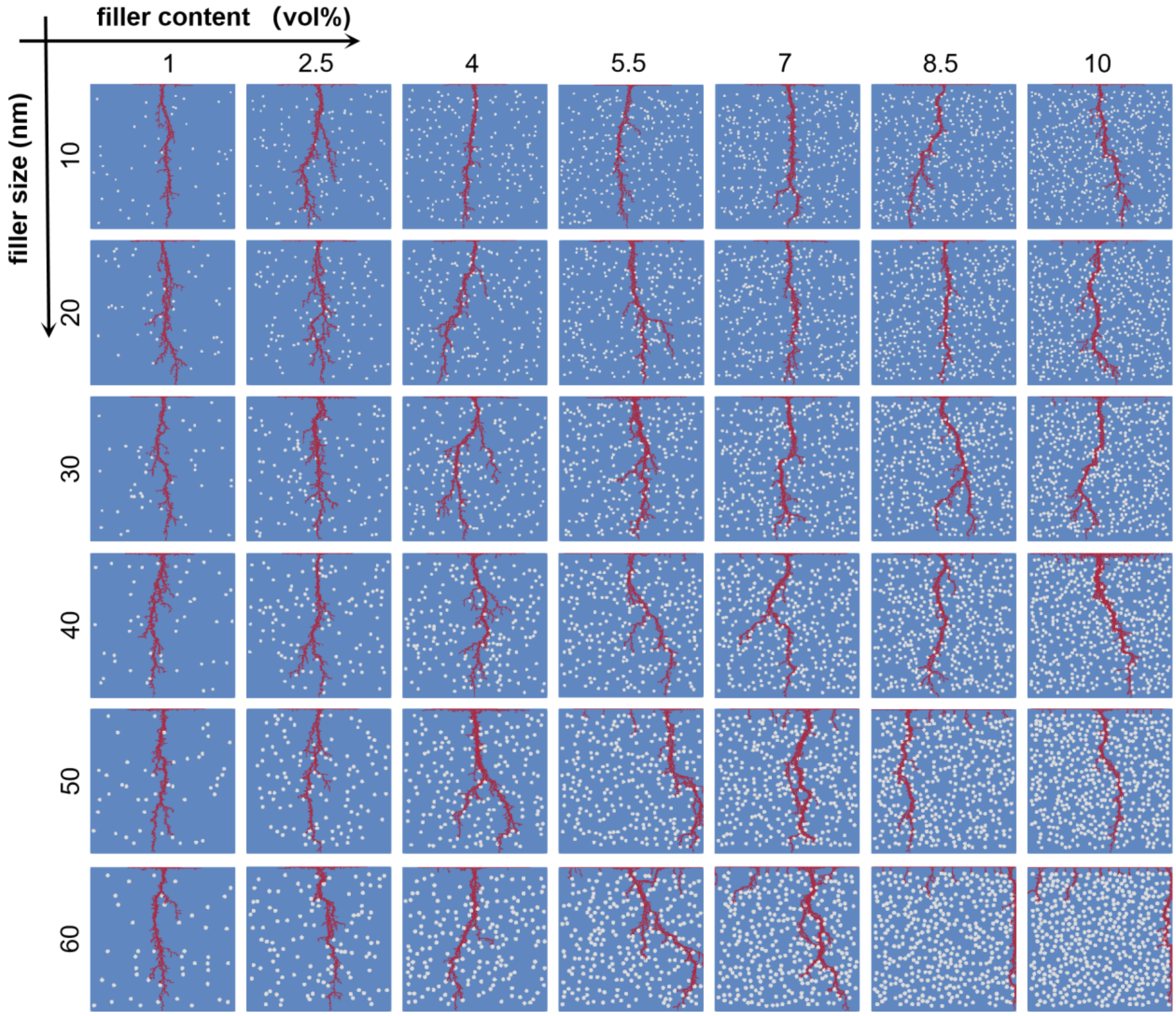

Figure S9. High-throughput stochastic breakdown simulation of PI/TiO_2 composites with different filler size and filler content.

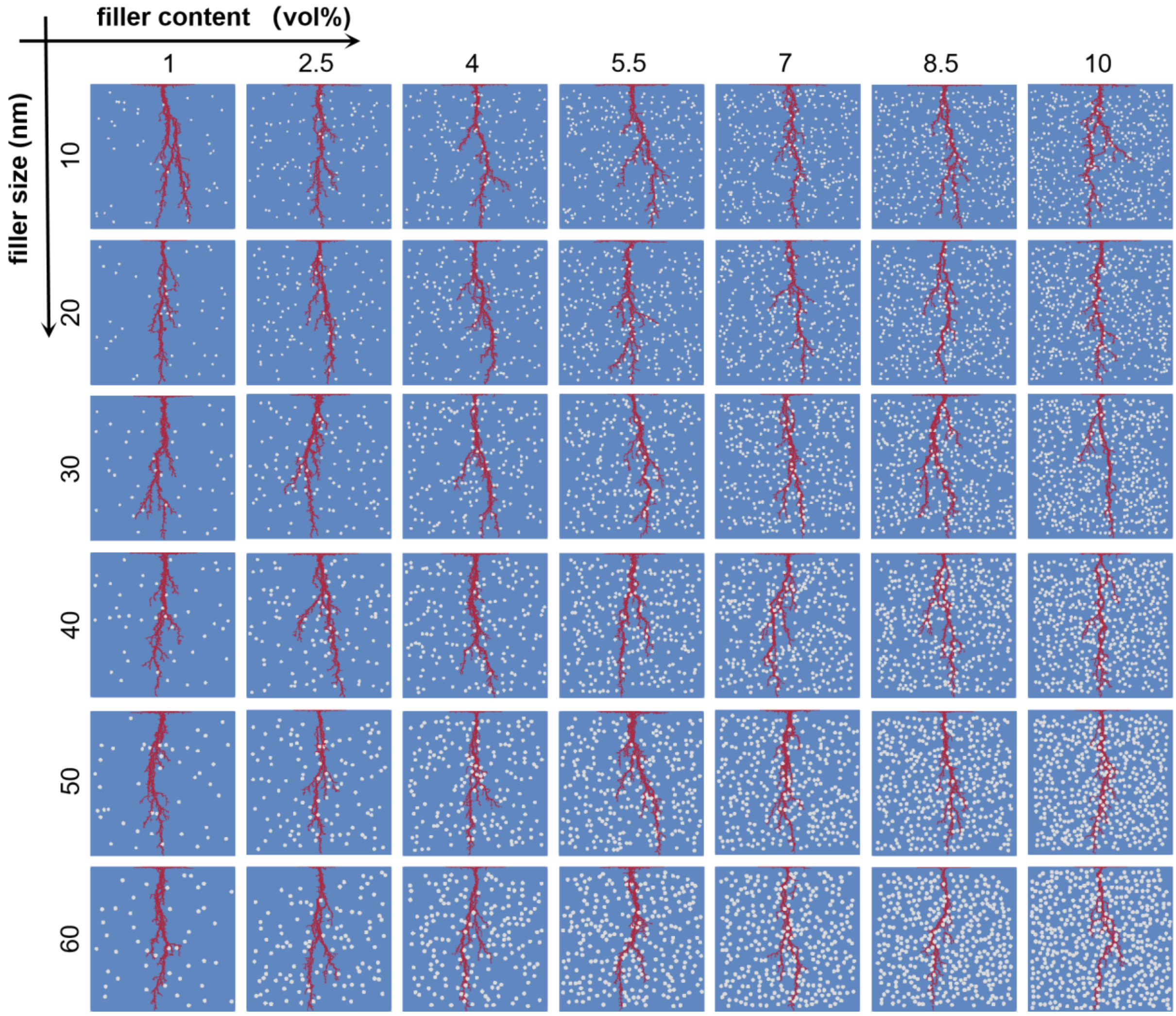

Figure S10. High-throughput stochastic breakdown simulation of PVDF/SiO_2 composites with different filler size and filler content.

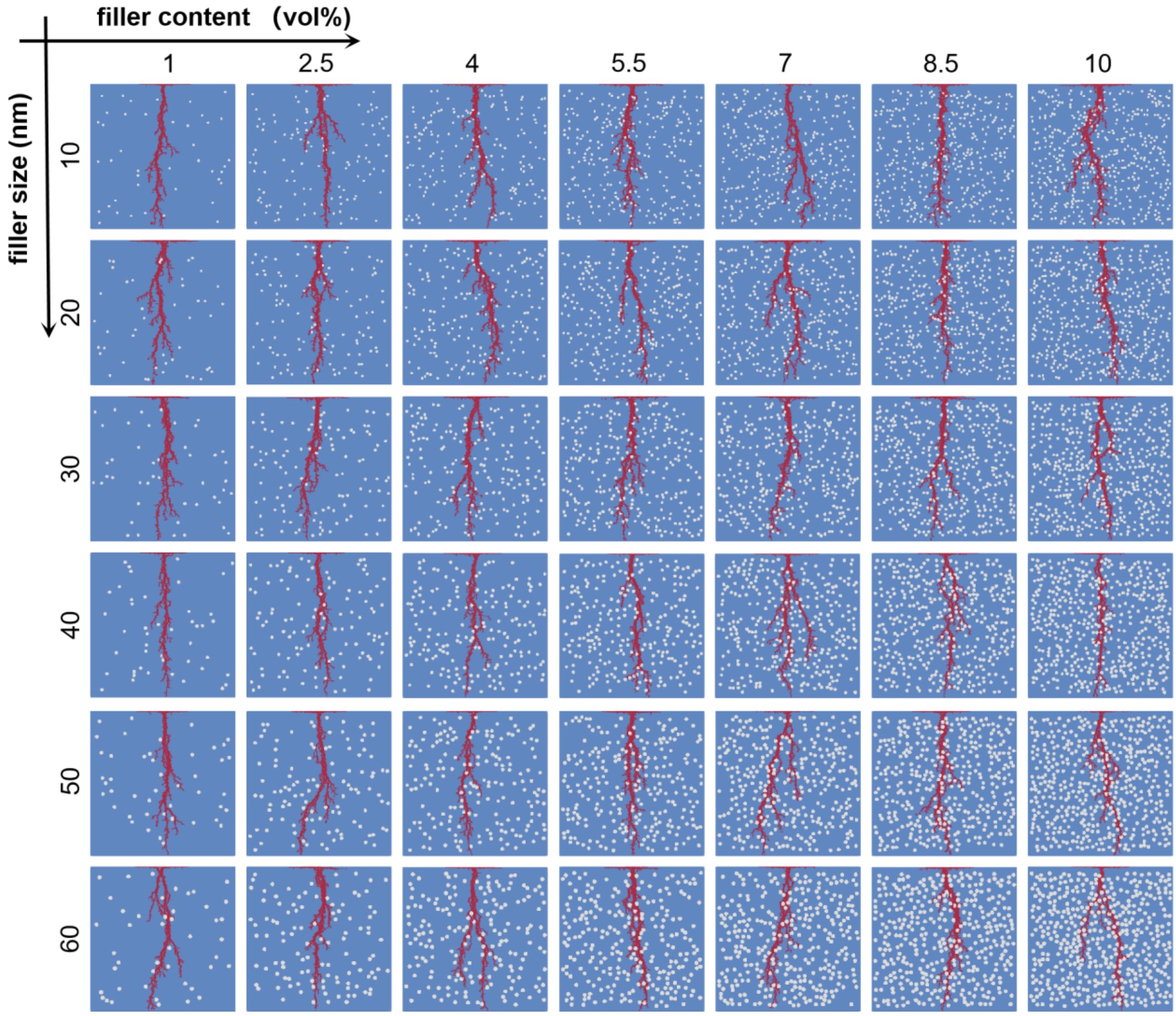

Figure S11. High-throughput stochastic breakdown simulation of PVDF/ZnO composites with different filler size and filler content.

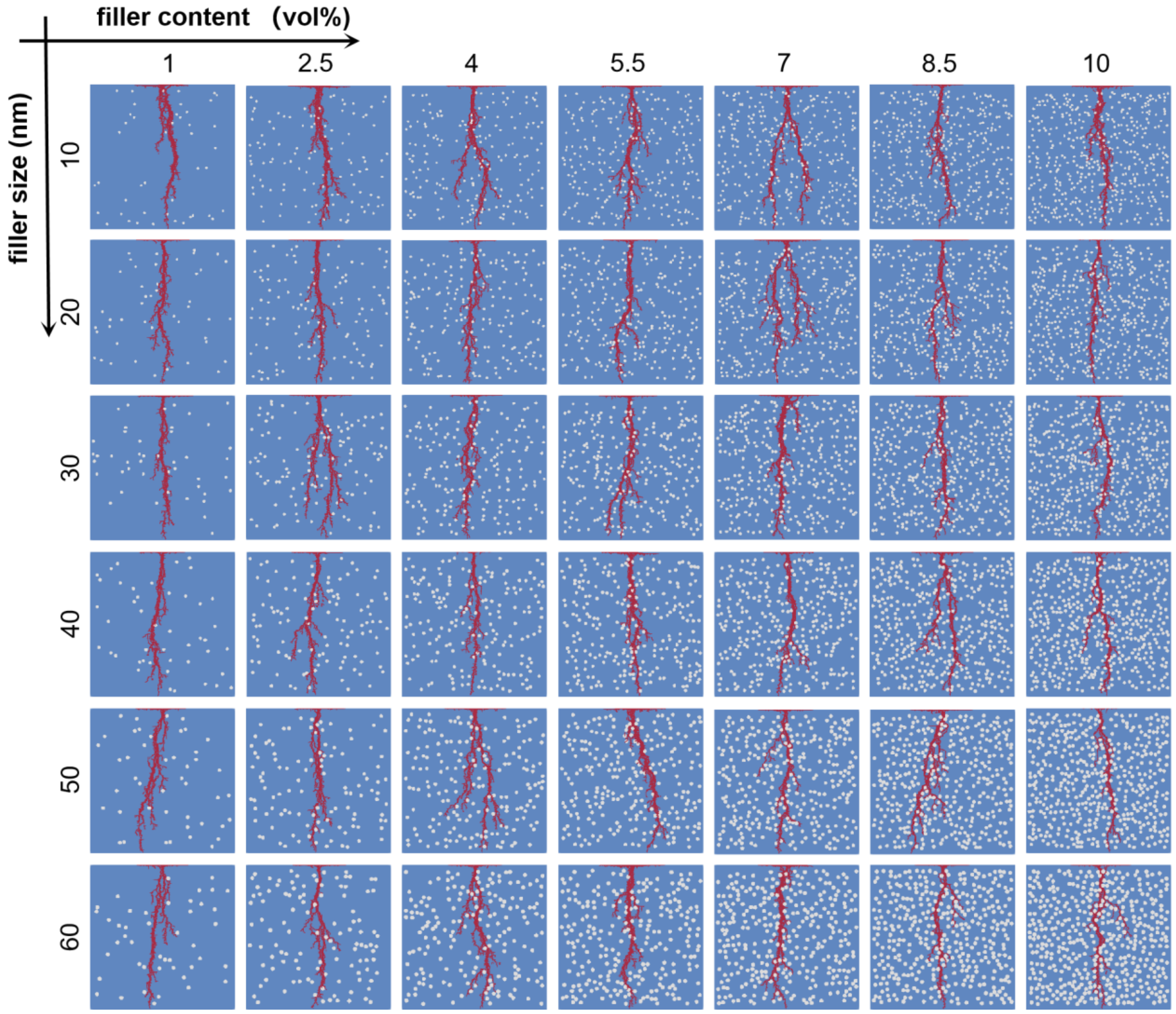

Figure S12. High-throughput stochastic breakdown simulation of PVDF/AlN composites with different filler size and filler content.

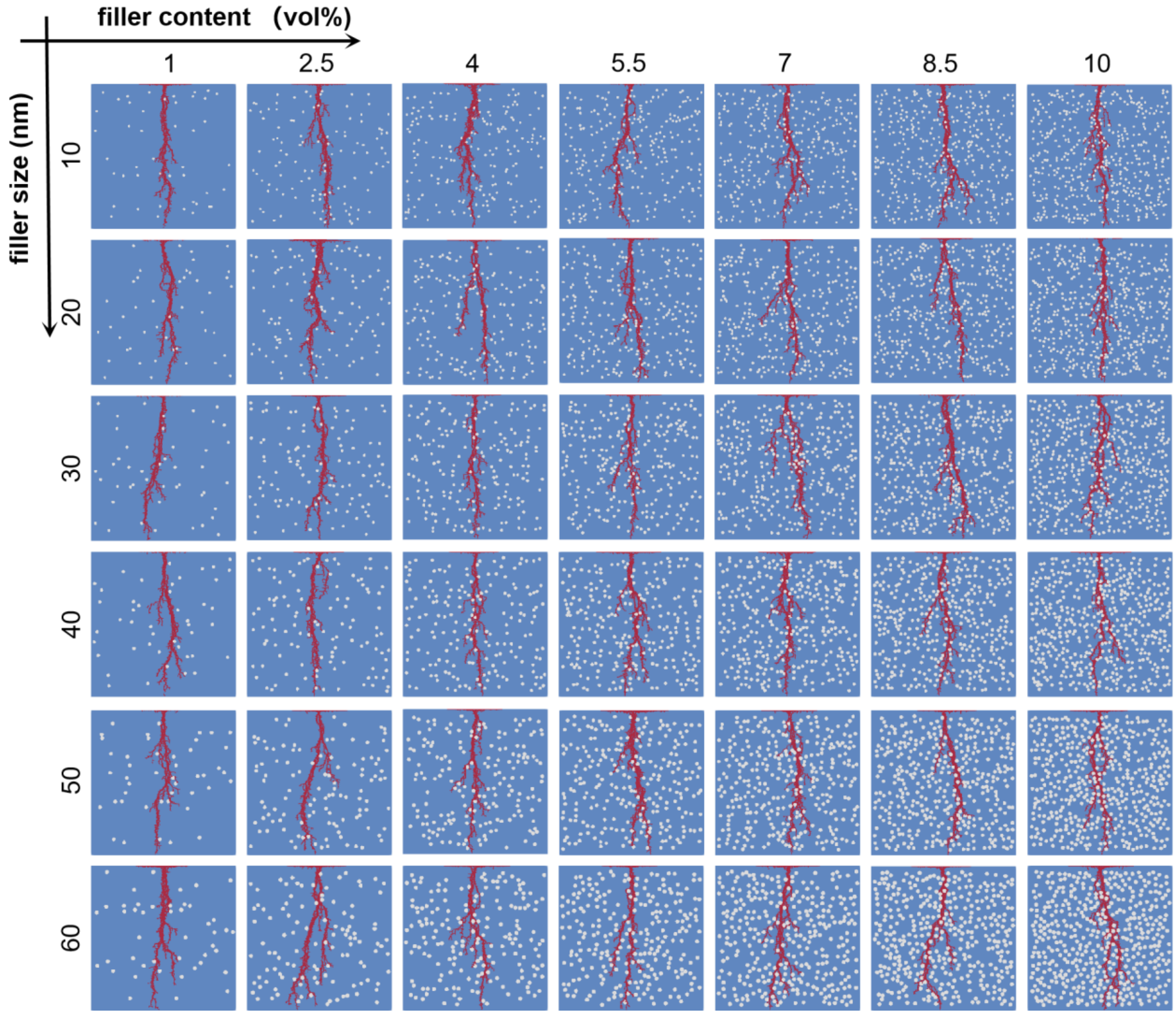

Figure S13. High-throughput stochastic breakdown simulation of PVDF/Al_2O_3 composites with different filler size and filler content.

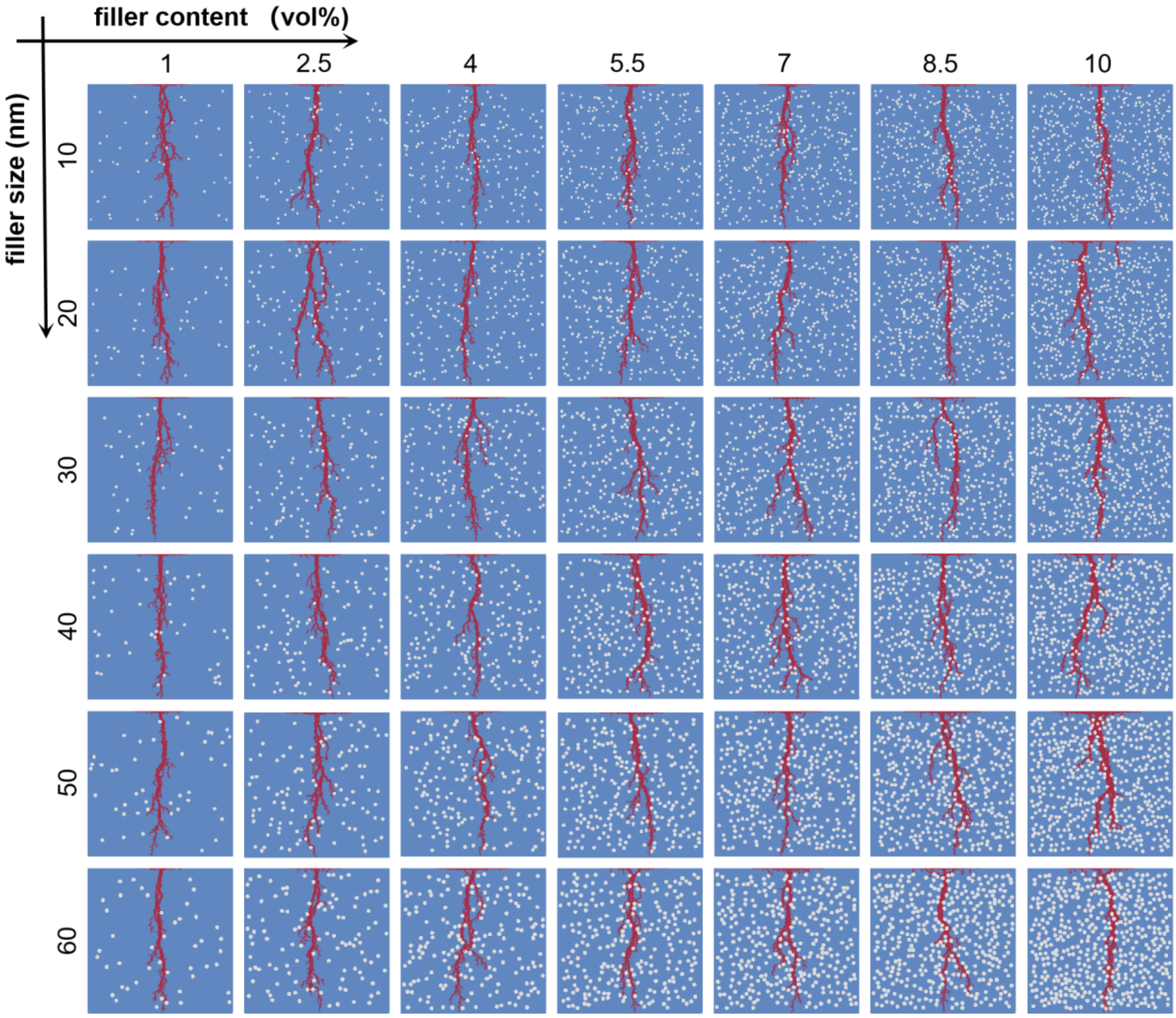

Figure S14. High-throughput stochastic breakdown simulation of PVDF/ZrO_2 composites with different filler size and filler content.

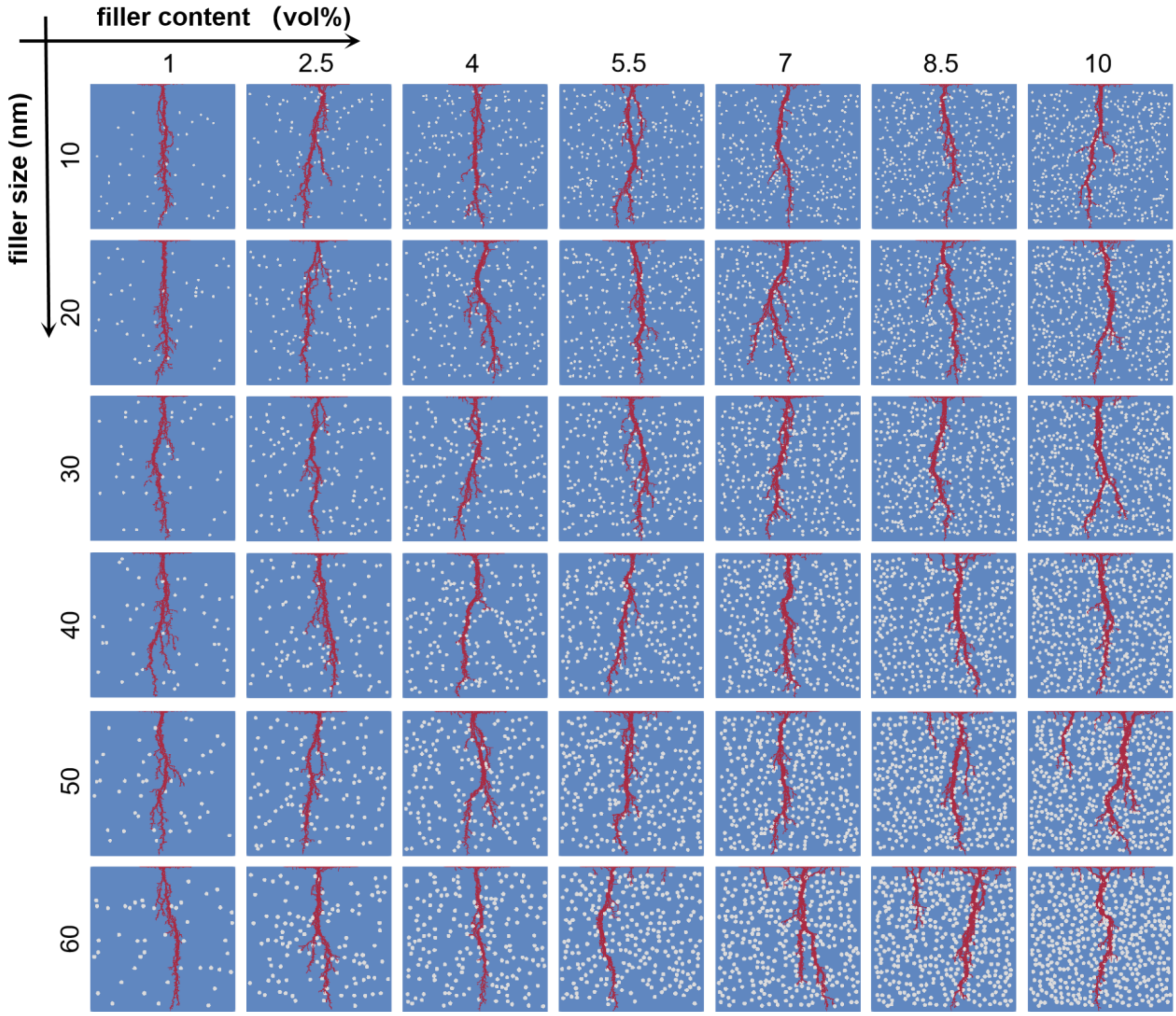

Figure S15. High-throughput stochastic breakdown simulation of PVDF/TiO_2 composites with different filler size and filler content.

Machine learning

Table S2. The coefficient of determination adjusted-R^2 in 1st round of least square regressions with only one variable and one of the 12 prototypical functions.

Descriptor	Adjusted-R^2 (only x_1 dielectric constant)	Adjusted-R^2 (only x_2 filler size)	Adjusted-R^2 (only x_3 filler content)
x	0.470	0.006	/
x^2	0.240	0.007	/
x^3	0.137	0.006	/
x^{-1}	0.668	0.004	0.008
x^{-2}	0.511	0.003	0.008
x^{-3}	0.424	0.002	0.008
$\ln(x)$	0.738	0.006	0.004
$\ln(x)^{-1}$	0.131	0.005	/
e^x	0.216	0.003	0.003
e^{-x}	0.671	0.001	0.009
$x^{1/2}$	0.625	0.006	0.002
$x^{-1/2}$	0.743	0.005	0.007

Table S3. The coefficient of determination adjusted-R^2 in 2nd round of least square regressions with two and three variables and corresponding best prototypical functions selected from Supporting Information Table S2, where x_1 takes the top three best prototypical functions, x_2 and x_3 take the best prototypical function.

Descriptor	Adjusted-R^2 (x_1 & x_2)	Descriptor	Adjusted-R^2 (x_1 & x_3)	Descriptor	Adjusted-R^2 (x_2 & x_3)	Descriptor	Adjusted-R^2 (x_1 & x_2 & x_3)
ln(x) & x^2	0.340	ln(x) & e^{-x}	0.090	x^2 & e^{-x}	/	ln(x) & x^2 & e^{-x}	0.042
e^{-x} & x^2	0.100	e^{-x} & e^{-x}	0.049			e^{-x} & x^2 & e^{-x}	0.015
$x^{-1/2}$ & x^2	0.153	$x^{-1/2}$ & e^{-x}	0.059			$x^{-1/2}$ & x^2 & e^{-x}	0.020

Table S4. The coefficient of determination adjusted-R^2 in the 3rd round least square regressions with consideration of interactions among descriptors. The total analytical function is

$$y = \beta_0 + \beta_1 (x_1)^{-1/2} + \beta_2 (x_2)^2 + \beta_3 e^{-(x_3)} + \beta_4 \ln(x_1)(x_2)^2 + \beta_5 \ln(x_1) e^{-(x_3)} + \beta_6 \ln(x_1)(x_2)^2 e^{-(x_3)} + e \quad (8)$$

	None	x_1	x_2	x_3	x_1 & x_2	x_1 & x_3	x_1 & x_2 & x_3	Adjusted-R^2
β	1.455	-0.089	0.00012	-0.156	0.000006	-0.061	-0.000019	0.778
P	0.001	0.001	0.001	0.001	0.001	0.007	0.083	F=295.488,P<0.001

Where P is used as an inspection standard in regression analysis, for x_1 & x_2 & x_3, it indicates that its influence on the dependent variable has no statistical significance when other variables remain unchanged, because the P value is greater than 0.05. Finally, the expression given by:

$$Y = 1.455 - 0.089(x_1)^{-1/2} + 0.00012(x_2)^2 - 0.156 e^{-(x_3)} + 0.000006 \ln(x_1)(x_2)^2 - 0.061 \ln(x_1) e^{-(x_3)} \quad (9)$$

Table S5. The polymer-based composites parameters used in the additional machine learning.[2-3]

	Filler dielectric constant	Filler size (nm)	Filler content (vol%)	$E_b^{composites}/E_b^{polymer}$	
				Experimental results	Machine learning
PVDF/SiO_2	4	30	5	1.31	1.52
PVDF/Al_2O_3	10	30	5	1.42	1.53
PVDF/TiO_2	59	30	3	1.17	1.33
PI/Al_2O_3	10	30	1	1.44	1.43
PI/Al_2O_3	10	30	3	1.48	1.53
PI/Al_2O_3	10	30	5	1.56	1.55
PI/Al_2O_3	10	30	7	1.62	1.55
PI/Al_2O_3	10	30	9	1.55	1.55
PI/HfO_2	17	30	1	1.38	1.43
PI/HfO_2	17	30	3	1.42	1.54
PI/HfO_2	17	30	5	1.53	1.54
PI/HfO_2	17	30	7	1.5	1.55
PI/HfO_2	17	30	9	1.46	1.55

Experimental section

Materials

Polyetherimide (PEI) was provided by PolyK Technologies. N-methylpyrrolidone (NMP) was produced by Tianjin Bailunsi Biotechnology Co., Ltd. Aluminium oxide (Al_2O_3, 30 nm) was obtained from Aladdin.

Preparation of PEI/Al_2O_3 composites

The PEI/Al_2O_3 composites loaded with different filler contents were fabricated by the physical blending method and the hot-pressing method as shown in Supporting Information Figure S16. A certain amount of Al_2O_3 fillers were taken and dispersed in NMP solution by sonication for 30 minutes. Then, the PEI particles were dissolved in the above dispersion in proportion, the magnetic stirrer temperature was set to 50 ℃, and stirring continued for 12 hours to obtain a uniform and stable suspension. The obtained mixed liquid was placed in a vacuum oven to take a vacuum, and the bubbles were discharged. Scraped and coated the solution on a glass sheet, then heated it 12 hours at 70 ℃. By hot-pressing the samples at 180 ℃ and 15 MPa, PEI/Al_2O_3 composites with a thickness of about 18 μm can be obtained.

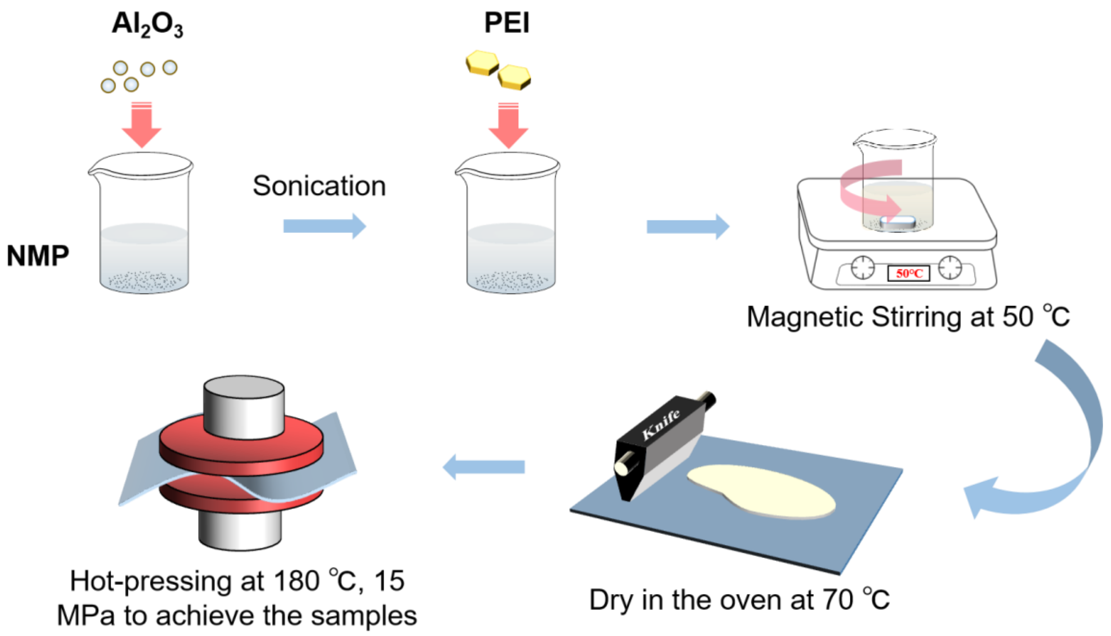

Figure S16. Synthetic flow chart of PEI/Al_2O_3 composites.

Characterization

The stochastic breakdown simulation and machine learning were implemented on a computer server. The phase change of the composites was tested using a Bruker D8 wide-angle X-ray diffraction scattering (XRD) instrument. Fourier Transform Infra-Red Spectrometer (FTIR) with a wave number range of 400-4000 cm^{-1} was used to characterize the chemical structures of the prepared PEI/Al_2O_3 composites. The number and resolution of scans in FTIR are 16 times and 4 cm^{-1}, respectively. Small Angle X-ray Scattering (SAXS) experiments were carried out at the beam line 1W2A of the Beijing Synchrotron Radiation Facility (BSRF). The storage ring was operated at 2.5 GeV with a current of about 80 mA. The charged coupled device type Mar165-CCD is used to collect 2D scattering patterns with a sample-to-detector distance in the beam direction of 1500 mm. The morphology of PEI/Al_2O_3 composites was characterized by the scanning electron microscope (SEM) of Helios Nano lab 600i. A Tecnai G^2 F20 transmission electron microscope (TEM) was used to detect the size of Al_2O_3. The Al electrodes (25 mm and 3 mm in diameter) were evaporated on two sides of the PEI/Al_2O_3 composites for the following electric measurements. The dielectric constant of the composites was measured by Concept 40 wide-band dielectric spectrometer at room temperature from 1 Hz to 10^7 Hz. The breakdown strength was investigated by an electric breakdown tester (YDZ-560) in silicone oil at a voltage ramp of 500 V/s. The test electrode for the breakdown strength was a ball-plate electrode, 8 data points were collected for each sample, and the two-parameter Weibull statistics distribution function was carried out.

Directional experimental simulation and testing

The Porod profiles of PEI/Al_2O_3 composites are shown in Supporting Information Figure S17. It can be seen that the curve has a certain degree of negative deviation. It is believed that the interaction between polymer molecular chains and Al_2O_3 fillers is responsible for the negative deviation in Porod plots. From the slope, with the increase of *v*, the curve of PEI/Al_2O_3 composites almost changes little, which again proves that the matrix and filler compatibility of each PEI/Al_2O_3 composite is good.

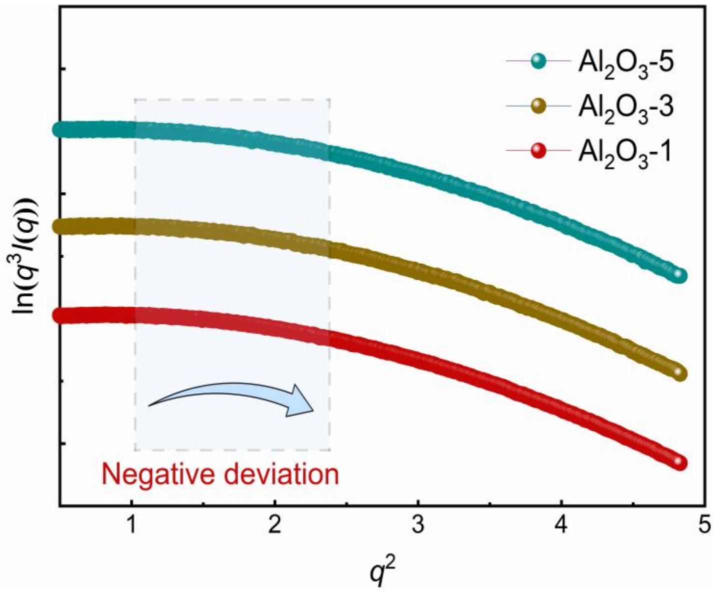

Figure S17. The Porod curves of PEI/Al_2O_3 composites.

Supporting Information Figure S18 is the SAXS diagram of the PEI/Al_2O_3 composites after Lorentz correction, which is used to detect the long cycle of semicrystalline polymer, generally appearing in the curve at the peak (highest point). However, it can be seen from Supporting Information Figure S18 that the curve keeps rising without a peak, indicating that the polymer is an amorphous polymer and that the fillers did not change the crystal state of the polymer.

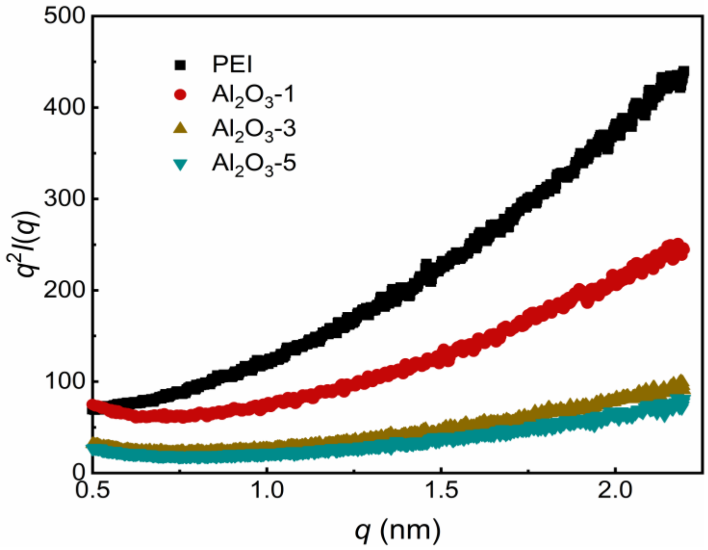

Figure S18. The SAXS diagram of the PEI/Al_2O_3 composites after Lorentz correction.

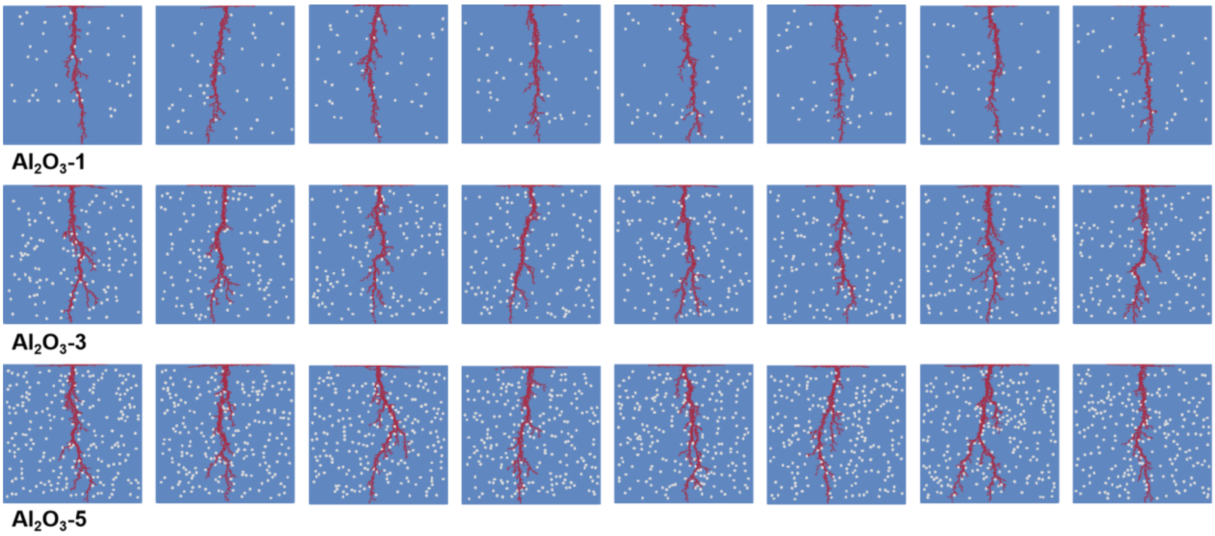

Figure S19. The stochastic breakdown simulation for PEI/Al_2O_3 composites.

The stochastic breakdown simulation of PEI/Al_2O_3 composites was carried out, and the results are shown in the Supporting Information Figure S19. The parameters used in the simulation are shown in Table S6. In each stochastic breakdown simulation process, the random distribution of fillers would have some influence on the breakdown path and E_b, so stochastic breakdown simulations were done 8 times for each PEI/Al_2O_3 composite. All simulated breakdown values are shown in Table S7. The simulation results of the 8 times breakdown of PEI/1 vol% Al_2O_3 composites were 456, 458, 459, 460, 462, 465, 466, and 467 kV/mm, respectively. No obvious variation can be attained between the 8 simulation results, which indicates the reliability of this simulation. The simulation results of the 8 times breakdown of PEI/3 vol% Al_2O_3 composites were 480, 481, 486, 488, 491, 493, 495, and 498 kV/mm from low to high, the results of these 8 simulations are also relatively close. The simulation results of the 8 times breakdown of PEI/5 vol% Al_2O_3 composites were 496, 499, 504, 505, 509, 509, 510, and 544 kV/mm. The simulation results changed from 496 to 544 kV/mm in these results, so it is necessary to calculate the breakdown value using the two-parameter Weibull distribution function for the simulation results.

Table S6. The composite parameters used in the simulation of this work.

Material	Dielectric constant	Breakdown strength (kV/mm)
Al_2O_3	10.0	800.0
PEI	4.0	363.0

Table S7. Stochastic breakdown simulation results of directional experiments.

The simulate E_b of PEI/1 vol% Al_2O_3 composite (kV/mm)	The simulate E_b of PEI/3 vol% Al_2O_3 composite (kV/mm)	The simulate E_b of PEI/5 vol% Al_2O_3 composite (kV/mm)
456.0	480.0	496.0
458.0	481.0	499.0
459.0	486.0	504.0
460.0	488.0	505.0
462.0	491.0	509.0
465.0	493.0	509.0
466.0	495.0	510.0
467.0	498.0	544.0

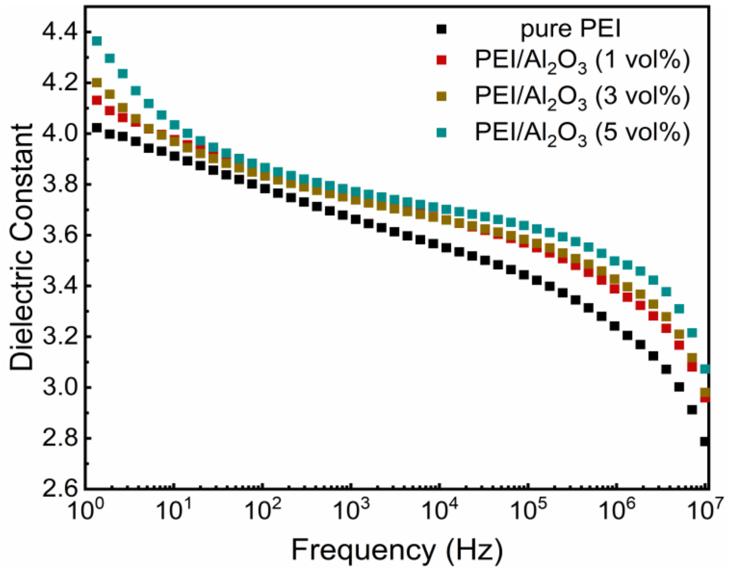

Figure S20. The dielectric constant for PEI/Al_2O_3 composites.

References

[1] J. J. Wang, X. Q. Ma, Q. Li, J. Britson, L.-Q. Chen, *Acta Materialia* **2013**, 61, 7591.

[2] D. Ai, H. Li, Y. Zhou, L. Ren, Z. Han, B. Yao, W. Zhou, L. Zhao, J. Xu, Q. Wang, *Advanced Energy Materials* **2020**, 10, 1903881.

[3] H. Li, T. Yang, Y. Zhou, D. Ai, B. Yao, Y. Liu, L. Li, L. Q. Chen, Q. Wang, *Advanced Functional Materials* **2020**, 31, 2006739.
